# Cross-species analysis reveals clonotypic T-cell remodeling underlying adrenal resistance to PD-1 blockade

**DOI:** 10.21203/rs.3.rs-11176760/v1

**Published:** 2026-10-16

**Authors:** Natalie Liu, Jay Uppuluri, Clemens Van Dongen, Annie Trinh, Jessica Schmeling, Omolola Olaleye, Cyrus Sholevar, Thant Zaw, Makan Karimzadeh, Marshall Lammers, Megan Purl, Amjad Mansour, Trung Tran, Joanne Xiu, W. Kimryn Rathmell, William Becker, Jinhwan Kim, Shuchi Gulati, Karleen Meiklejohn, Michael Corwin, Arta Monjazeb, Claire Graves, William Murphy, Sean Judge, Michael Campbell, Anthony Zamora, Robert Canter

## Abstract

Although clinical data suggest that adrenal metastases from solid tumors are less responsive to immune checkpoint inhibition (ICI) compared to other organ sites, little is known about the adrenal immune microenvironment’s role in metastatic growth and response to immunotherapy. Using a novel clinical and genomic database of renal cell carcinoma (RCC) patients, matched human adrenal and PBMC specimens, and orthotopic murine tumors, we identified an adrenal immune state enriched for PD-1+ activated, effector memory, and tissue-resident T cells rather than an immune desert. Paired single-cell RNA (scRNA) and T cell receptor (TCR) sequencing of benign adrenal tissue and peripheral blood showed reproducible adrenal enrichment of activation, hypoxia, TNF-NF-κB, and mTORC1 programs, reduced repertoire diversity, and tissue-dependent state remodeling between shared clonotypes. In Renca and MC-38 tumor models, adrenal tumors showed attenuated and less durable responses to PD-1 blockade compared to subcutaneous tumors, and Renca adrenal tumors failed to mount the treatment-associated effector memory response observed subcutaneously. Overall, our cross-species analysis identifies the adrenal gland as a distinct immune compartment with a unique population of adrenal PD-1+ T cells with resistance to ICI treatment despite an activated, effector phenotype.

**Summary:** Using clinical data, human adrenal and adrenal metastasis specimens, and orthotopic murine models, we uncovered a previously unrecognized mechanism of resistance to PD-1 blockade in the adrenal gland. Adrenal metastases in human subjects and mouse models were refractory to immune checkpoint inhibition despite the presence of abundant, functional PD-1+ effector T cells, Strikingly, paired TCR sequencing revealed distinct and previously unrecognized T cell clonotypes enriched within the adrenal gland, suggesting that adrenal resistance is not simply a consequence of T cell exclusion or dysfunction, but reflects a unique tissue-specific immune ecosystem that shapes T cell recruitment, clonality, and response to PD-1 blockade. Together, these multispecies findings identify the adrenal gland as a uniquely immunobiologically distinct site of immune checkpoint resistance.

## Introduction:

The adrenal glands are central regulators of stress responses, metabolism, electrolyte homeostasis, and sympathetic nervous system function. Yet their immune function and architecture remain poorly defined. They have historically been described as immune privileged, primarily because the preservation of endocrine function is essential and excessive local inflammation could be deleterious [Deng et al 2025, Bedogni et al 2020]. This designation has long been considered to stem from evolutionary mechanisms serving to prevent autoimmunity and protect essential organ function, similar to other immune-privileged organs such as the eye and reproductive tissues [Stein-Streilein et al 2014, Arck et al 2014, Benhar et al 2012, Apps et al 2008]. However, few studies have comprehensively examined the immunobiology of the adrenal gland or its tumor microenvironment (TME) nor how it responds in the setting of benign and malignant adrenal neoplasia [Chen et al 2026, Liu et al 2026]. Thus, there is an important clinical need to understand adrenal-specific immune regulation, especially in light of accumulating studies demonstrating that the adrenal glands are sanctuary sites for metastatic cancer [Nguyen et al 2018, Cohen at al 2021, Siemer et al 2004].

Immune checkpoint inhibition (ICI) targeting the programmed cell death-1 (PD-1) and programmed cell death ligand-1 (PD-L1) pathway has resulted in durable responses across multiple malignancies, including melanoma, non-small cell lung cancer (NSCLC), and renal cell carcinoma (RCC) [Patel et al 2023, Machado et al 2026, Chouieri et al 2024]. PD-1 blockade can reinvigorate antigen-experienced tumor-reactive T cells, but many patients either fail to respond or ultimately develop resistance [Jenkins et al 2018, Wolchok et al 2025, Kluger at el 2020, Sharma et al 2017, Mariniello at al 2025, Vlachostergios et al 2020, Lotfi et al 2026]. Established correlates of response include tumor antigenicity, intact antigen presentation and interferon signaling, and pre-existing intratumor T cell populations capable of responding to immune checkpoint blockade [Naing et al 2026, Tumeh et al 2014, Hutchins et al 2020, Ayers et al 2017, So et al 2023, Yi et al 2018, Bevins et al 2022, Koh et al 2022]. These determinants, however, do not fully explain why lesions within the same patient can respond differently according to anatomic site.

Recently, clinical data have highlighted that patients with adrenal metastases, from RCC and lung carcinoma among others, experience significantly worse outcomes than patients with metastases at other sites, especially when treated with ICI [Borgers et al 2021, Billon et al 2024, Vaishampayan et al 2020]. In fact, retrospective analyses have revealed that adrenal metastases are associated with shorter progression-free survival and lower overall survival among patients treated with ICI targeting PD-1 or PD-L1. Adrenal metastases often demonstrate resistance to PD-1 blockade even in patients whose tumors respond favorably at other sites. These observations raise a central mechanistic question: does adrenal resistance to ICI reflect a paucity of antitumor effector T cells, or does the adrenal microenvironment qualitatively alter the state and treatment responsiveness of infiltrating and local T cells? Addressing the underlying mechanisms of the adrenal "sanctuary site" could improve outcomes for patients with RCC, lung cancer, melanoma, and other malignancies prone to adrenal metastasis.

Most mechanistic models of PD-1 resistance emphasize tumor-cell-intrinsic defects, T cell exclusion, suppressive myeloid or stromal populations, or terminal T cell dysfunction [Benci et al 2016, Mendal et al 2025, Wang et al 2025, Chen et al 2025]. In contrast, comparatively little is known about organ-specific immune programs that may shape otherwise antigen-experienced T cells [Nowicki et al 2018]. Due to the adrenal glands' specialized role in endocrine and metabolic functions, defining their baseline immune microenvironment state and adrenal-resident T cells' response to PD-1 blockade may reveal a distinct form of tissue-associated resistance.

Given this gap in knowledge, we sought to define the adrenal immune microenvironment and test whether this specific anatomic site modifies response to PD-1 blockade by combining clinical outcome analyses, prospective human tissue profiling, paired scRNA-seq and TCR-seq, and orthotopic murine tumor models. Across species, we did not observe an exclusion of T cells, but an abundance of PD-1+ effector/memory and tissue-resident T cells with a distinct activation- and tissue-conditioning-associated transcriptional program. In mice, the adrenal site attenuated and reduced the durability of anti-PD-1 responses and failed to support the treatment-associated effector memory expansion observed in subcutaneous tumors. These findings identify adrenal tissue as a distinct immune compartment and suggest that the presence of PD-1+ T cells is insufficient to predict benefit from PD-1 blockade. We aimed to uncover mechanisms driving adrenal PD-1 resistance by defining the molecular and cellular adrenal immune milieu, further supporting that insights gained from this study may extrapolate to other endocrine-related sanctuary sites and help unlock potentially novel mechanisms of immunotherapy resistance. Our results indicate the presence of a biologically distinct adrenal microenvironment, characterized by robust infiltration of activated PD-1+ T cells, and validate that adrenal metastases exhibit intrinsic resistance to PD-1 blockade that is driven by local, tissue-specific factors which blunt effective immune reinvigoration. This discovery underscores the unique, organ-specific immunobiology of the adrenal gland and adrenal metastases across species. This work challenges the prevailing assumption that PD-1 resistance is solely tumor-cell intrinsic or uniformly systemic, instead highlighting the adrenal glands as sites of immune resistance that can undermine ICI efficacy. Defining this unique immune milieu could show a novel mechanism of resistance to ICI with implications to develop therapies to reverse this organ-specific phenomenon and improve ICI efficacy.

## **Results**

*Clinical data identify adrenal metastasis as a site of resistance to immune checkpoint inhibition*

To validate the adrenal glands as sites of organ specific resistance to ICI in RCC, we first sought to evaluate tissue specimens in a retrospective cohort of RCC patients with adrenal metastases treated over an 8-year period at our institution (**Table 1**). Matched primary tumor, adrenal metastases, and non-tumor adrenal samples from this cohort were collected and analyzed for PD-1 expression by immunohistochemistry (IHC,) as shown in **Figure 1A**. Notably, both adrenal metastasis and non-tumor adrenal samples exhibited significantly higher PD-1 staining compared to primary RCC tumors (H score: 82.6±51.6 vs 27.5±47.1 vs 8.87±20.8, $p<0.05$) (**Figure 1B**). Furthermore, when this retrospective cohort was stratified based on exposure to ICI prior to adrenal metastasectomy, we found that PD-1 was significantly decreased in patients who received ICI compared to those who were ICI naive (H score: 5.48±9.10 vs 31.71±42.5, $p<0.05$) (**Figure 1C**). In the subpopulation of patients treated with ICI (pembrolizumab, nivolomab, atezolizumab) prior to adrenal metastasectomy (N=5), we then assessed changes in tumor size in adrenal metastatic lesions compared to extra-adrenal metastatic lesions (bone, lung, liver) in the same patients. Notably, we observed that adrenal metastatic lesions exhibited higher cumulative tumor growth while on ICI compared to the extra-adrenal metastatic lesions (+4.41±3.51 cm vs +0.50±0.00 cm, $p<0.05$) (**Figure 1D**), which is consistent with adrenalspecific ICI resistance. **Figure 1E** shows a representative RCC patient pre and post ICI who experienced increased growth of an adrenal metastatic lesion from 10 mm to 23 mm compared to tumor shrinkage in a liver lesion which decreased from 24 mm to 10 mm over the same treatment period as demonstrated on matched CT imaging (**Figure 1E**).

As our retrospective cohort was limited by small sample size, we then expanded our investigation of clinical outcomes using a larger comprehensive patient database. We queried the CODEai$^{TM}$ clinical and genomic database from Caris Life Sciences [Abraham et al 2025] and identified 132 RCC patients with adrenal metastasis and 1,327 RCC patients with extra-adrenal metastasis similar to our previous IHC cohort (bone, lung, liver). When stratifying these two groups by ICI treatment or no ICI treatment, we found that ICI treatment did not result in a survival benefit in the RCC adrenal metastasis cohort ($p=0.677$) (**Figure 1F**). Conversely, the RCC extra-adrenal metastasis cohort demonstrated significantly longer overall survival among patients who received ICI compared to those that did not ($p<0.001$) (**Figure IG**). These results support resistance to ICI in RCC patients with adrenal metastasis and identify PD-1 expressionas a potential important immunophenotypic characteristic which can be modified post ICI.

*Adrenal metastases and non-tumor adrenal tissue contain abundant activated PD-1+ T cells*

To define the immune context of adrenal metastasis prospectively, we analyzed matched PBMCs, adrenal metastasis, and adjacent non-tumor adrenal tissue from four patients undergoing adrenal metastasectomy (**Figure 1A**, Table 2). Utilizing multi-color flow cytometry we observed that the lymphocyte populations of both adrenal metastasis and non-tumor adrenal tissue had a significantly higher frequency of CD3+ T cells compared to PBMCs (77.0±19.0 and 75.4±12.3

versus 44.3± 9.0; p<0.05), and adrenal metastasis contained a greater T cell fraction among live CD45+ cells compared to PBMCs (82.9±3.0 vs 47.0±9.4, p<0.05) (**Figure 2B**). While PBMCs expressed greater CD4+ T cells compared to CD8+ T cells (67.9±7.2 vs 28.8±7.1, p<0.05), adrenal metastasis and non-tumor adrenal tissue showed similar proportions of CD4+ and CD8+ T cells (**Supplemental Figure 1A**). Furthermore, similar to our IHC data, we observed that PD-1 expression was markedly higher on T cells of adrenal metastasis and non-tumor adrenal tissue compared to PBMCs on both CD4+ T cells (82.9±8.7 and 63.9±12.3 vs 9.1±3.0, p<0.05) and CD8+ T cells (72.8±28 and 56.4±12.7 vs 15.7±9.7, p<0.05) (**Figure 2C**). Additionally, expression of CD69 (marker of early activation and tissue lymphocyte retention) was also significantly higher in adrenal metastasis and non-tumor adrenal compared to PBMCs on both CD4+ T cells (87.0±3 and 72.4±26.8 vs 5.1±3.8, p<0.05) and CD8+ T cells (81.2±20.7 and 72.5±25.9 vs 6.7±4.1, p<0.05) (**Figure 2D**).

We then sought to evaluate the immune microenvironment of the non-tumor adrenal gland in non-cancer patients. As the excision of normal adrenal glands is not an indication for elective surgery, we instead studied matched PBMCs and non-tumor adrenal tissue from a prospective cohort of patients (N=25) undergoing adrenalectomy for benign incidentalomas (**Supplemental Table 1**), hypothesizing that this would be the closest achievable model of the native human adrenal gland immunobiology. We found that adrenal lymphocytes showed a significantly higher predominance of T cells compared to PBMCs (68.6±0.0 vs 48.1±0.0, p<0.05) although there was no significant difference when looking at the T cell proportion of all live CD45+ cells (**Figure 3A**). Moreover, the ratio of CD4+ T cells to CD8+ T cells was significantly inverted in adrenal tissue compared to PBMCs with non-tumor adrenal showing a significantly larger CD8+ T cell subpopulation compared to CD4+ T cells (50.2±14.0 vs 32.5±11.0, p<0.05) (**Figure 3B**). Similar to our analysis of adrenal metastases patients, PD-1 expression in benign adrenalectomy patients was also significantly higher in adrenal tissue compared to PBMCs on both CD4+ T cells (58.7±20.1 vs 13.2±9.7, p<0.05) and CD8+ T cells (72.3±13.2 vs 16.8±12.8, p<0.05) (**Figure 3C**), suggesting that higher expression of PD-1 on adrenal gland T cells is not limited to patients with metastasis. Similar to our adrenal metastasis cohort, CD69 expression was also significantly higher in benign non-tumor adrenal tissue compared to PBMCs on both CD4+ T cells (73.9±19.2 vs 5.5±4.0, p<0.05) and CD8+ T cells (83.5±16.4 vs 7.5±6.1, p<0.05 (**Figure 3D**). Furthermore, there was a notable increase in the co-expression of both PD-1 and CD69 in the adrenal tissue on both CD4+ T cells (46.2±20.3 vs 0.8±2.4, p<0.05) and CD8+ T Cells (63.6±15.6 vs 1.9±4.9, p<0.05) suggesting that the adrenal PD-1+ T cells had an activated phenotype and were not exhausted (**Figure 3E**). As CD69 is both a marker for T cell activation and tissue lymphocyte retention, we examined other activation markers to better determine if adrenal T cells were functional or hypofunctional. While HLA-DR expression is classically seen on antigen-presenting cells, it is also expressed on activated T cells [Nguyen et al 2022, Long et al 2025]. When compared to PBMCs, we observed significantly higher HLA-DR expression on adrenal CD4+ T cells (40.1±16.7 vs 11.3±12.5, p<0.05) and

CD8+ T cells (52.6±22.1 vs 20.2±15.8, $p<0.05$) (**Figure 3F**). Additionally, we found increased co-expression of PD-1 and HLA-DR in adrenal tissue on both CD4+ T cells (21.2±17.8 vs 9.17±11.1, $p<0.05$) and CD8+ T cells (36.9±21.5 vs 17.7±15.5, $p<0.05$) (**Figure 3G**). To further assess functionality in adrenal T cells, we measured cytokine secretion in ex vivo adrenal and PBMC T cells stimulated with phorbol myristate acetate (PMA). We found that adrenal T cells expressed significantly higher IFNγ among both CD4+ T cells (32.9±15.6 vs 8.6±6.2, $p<0.05$) and CD8+ T cells (58.4±9. vs 19.4±6.2, $p<0.05$) (**Figure 3H**). Adrenal T cells also displayed significantly higher TNFα cytokine secretion, although this was limited to the CD8+ T cell subpopulation (66.3±11.1 vs 24.2±8.6, $p<0.05$) (**Figure 3I**). Likewise, adrenal T cells also displayed significantly higher IL-2 on CD8+ T cells (40.0±4.7 vs 12.2±1.6, $p<0.05$) (**Figure 3J**). Taken together, these prospectively collected data highlight the existence of a robust population of T cells in adrenal tissue that expresses PD-1. Moreover, the significantly increased CD69 and HLA-DR on PD-1+ T cells, and significantly greater cytokine secretion by adrenal T cells (especially CD8+ T cells) suggest that these adrenal T cells remain functional rather than exhausted or dysfunctional despite PD-1 expression.

*The adrenal T-cell compartment is enriched for effector-memory and tissue-resident phenotypes*

We further characterized T cell differentiation in matched PBMC and non-tumor adrenal samples from the benign adrenalectomy cohort (**Supplemental Table 1**). We first identified a significantly higher frequency of effector memory (CD45RA-CCR7-) T cells present among adrenal CD4+ T cells compared to PBMCs (68.8±17.6 vs 27.4±15.3, $p<0.05$) (**Figure 4A**). Similarly, we observed a significantly higher frequency of effector memory T cell subsets among adrenal CD8+ T cells compared to PBMCs (82.8± 9.53 vs 41.2±24.1, $p<0.05$) (**Figure 4B**). To elucidate if these were tissue-resident adrenal T cells, we evaluated tissue residency markers including CD69 and CD103, observing significantly increased co-expression of these markers on both adrenal CD4+ T cells (10.5±9.3 vs 0.02±0.0, $p<0.05$) and CD8+ T cells (32.0±13.5 vs 4.3±4.3, $p<0.05$) (**Figure 4C**). We then examined the expression of PD-1 on these T cell subsets. Notably, we observed increased PD-1 expression on all adrenal T-cell subsets compared to PBMCs, with PD-1 expression the highest on effector memory CD4+ T cells (78.3±17.3 vs 37.7±16.9, $p<0.05$) (**Figure 4D**) and effector memory CD8+ T cells (82.8±9.5 vs 41.2±24.1, $p<0.05$) (**Figure 4E**). As shown in Figure 4F, we also observed significantly higher PD-1+ expression on both adrenal tissue resident CD4+ T cells (89.5±9.7 vs 11.5±18.9, $p<0.05$) and CD8+ T cells (8.2±21.4 vs 33.3±32.7, $p<0.05$). These findings challenge the view that the adrenal glands are immune deserts and instead identify a resident, phenotypically activated T cell population with high PD-1 expression.

*Paired single-cell profiling reveals an activation and tissue remodeling program across CD4 and CD8 T cell lineages*

Given the limitations of IHC and flow cytometry for in-depth phenotypic and functional immune analyses, we expanded upon our data using paired single-cell RNA sequencing and T cell receptor (TCR) sequencing on non-tumor adrenal tissue and PBMCs from our benign adrenalectomy donors (eight samples; **Supplemental Table 2**). Integrated reclustering of 15,060 T cells from non-tumor adrenal tissue and PBMCs identified 20 transcriptionally distinct clusters, which were assigned to 12 T cell states based on cluster-enriched genes and canonical T cell state markers (**Supplemental Figure 2A-D**). Similar to our flow cytometry analysis, comparison of matched adrenal and PBMC samples revealed a pronounced shift in T cell state composition (**Figure 5A,B; Supplemental Figure 2E,F**). Adrenal T cells were enriched for an activation-associated state and, to a lesser extent, an effector memory (TEM) state, whereas matched PBMCs contained larger proportions of naïve, central memory (TCM) and cytotoxic T cell states. Mean adrenal-minus-PBMC differences were +46.3±7.2 percentage points for the activation-associated state and +14.6±4.4 points for TEM, compared with -25.7±6.5 for naïve, -19.4±5.3 for TCM, and -20.0±11.9 for the cytotoxic state (mean±SEM; **Figure 5C**). All five shifts were directionally concordant across the four paired donors (**Supplemental Figure 2F**), supporting reproducible remodeling of the T cell state composition in adrenal tissue.

Given the divergent phenotypes between adrenal and PBMC derived T cells, we next tested whether distinct tissue-associated transcriptional programs would be found in T cells from both locations. After aggregating T cell expression within each sample and statistically controlling for inter-individual differences among the four paired donors, adrenal T cells showed reproducible transcriptional differences relative to matched circulating T cells. Adrenal T cells showed increased expression of PDCD1 and genes associated with immediate-early activation, stress-response, and tissue-conditioning genes, including NR4A1, AREG, DUSP4, EGR2, HSPA1A, HSPA1B, EGR2, and EPAS1. In contrast, PBMC derived T cells preferentially expressed genes associated with circulating naïve and memory states, including SELL, TCF7, and LEF1, as well as cytotoxic-associated genes including CX3CR1, FGFBP2, and GNLY (Figure 5D, E). Consistent with these transcriptional differences, preranked gene-set enrichment analysis identified relative enrichment of TNF-NF-κB signaling, hypoxia, mTORC1 signaling, apoptosis, cellular responses to lipid, and αβ T cell activation in adrenal T cells. Conversely, PBMC derived T cells were enriched for RNA translation, ribosome and ribonucleoprotein-complex biogenesis, and mitochondrial gene-expression programs (**Figure 5F**). Together, these findings indicate that adrenal based T cells exhibit a reproducible activation- and tissue-conditioning-associated program distinct from circulating T cells.

Because the global comparison could be influenced by different CD4-to-CD8 ratios, we repeated the analysis within each T cell lineage. Independent CD4+ and CD8+ reclustering recapitulated the tissue-associated remodeling observed in global T cell analysis (**Supplemental Figure 3A-C**). In both T cell lineages, adrenal tissue contained larger activation-association populations, whereas PBMC CD4+ T cells were enriched for naïve and central memory states and PBMC CD8+ T cells retained a larger cytotoxic compartment (**Supplemental Figure 3B,C**). Lineage-resolved expression analysis likewise showed higher NR4A1, AREG, HSPA1A, HSPA1B, and PDCD1 in adrenal CD4+ and CD8+ T cells. PBMC CD4+ T cells expressed more SELL, TCF7, LEF1, and IL7R, whereas PBMC CD8+ expressed more CX3CR1, FGFBP2, GNLY, and GZMB (**Supplemental Figure 3D,E**). The adrenal-associated transcriptional phenotype was therefore evident within both major T cell lineages and was not explained solely by altered CD4-to-CD8 frequencies.

Pathway-level analysis further identified both shared and lineage-specific features of adrenal remodeling (**Supplemental Figure 3F,G**). Both adrenal CD4+ and CD8+ T cells showed enrichment of inflammatory, activation, hypoxia, and mTORC1-associated transcriptional programs relative to matched PBMCs, whereas translational and mitochondrial programs were preferentially represented in circulating T cells. Donor-paired KEGG scoring showed increased cytokine-cytokine receptor interaction scores in adrenal tissue in all four donors and higher antigen-processing and presentation scores in three of four donors in each T cell lineage. In contrast, ribosome, oxidation phosphorylation, glycolysis/gluconeogenesis, and NK-cell-mediated cytotoxicity scores decreased in adrenal tissues across all four donors in both lineages. TCR-signaling scores were lineage dependent, increasing in three of four adrenal CD4+ T cell samples but decreasing in all four adrenal CD8+ T cell samples (**Supplemental Figure 3G**). Together, these data define coordinated tissue conditioning across both lineages while revealing lineage-specific changes in individual signaling programs.

*Orthotopic murine tumors recapitulate site-dependent resistance to PD-1 blockade*

To better assess mechanisms of adrenal resistance to ICI in vivo, we turned our attention to the development of a novel model of orthotopic tumor implantations into murine adrenal glands. We used the murine renal cell carcinoma tumor line Renca which is syngeneic in BALB/c mice and partially responsive to PD-1 blockade in vivo [Pham et al 2022, Fabian et al 2017, Kunimura et al 2020, O'Shaughnessy et al 2018] and MC-38 (a colorectal carcinoma tumor line) which is syngeneic to C57BL/6 and typically highly responsive to PD-1 blockade [Jin et al 2022, Pauken et al 2025, Berg-Larsen et al 2022] (**Figure 6A**). Using high resolution ultrasound imaging, we orthotopically injected tumor cells from both lines directly into the adrenal glands of mice (**Figure 6B**) and compared anti-tumor effects of PD-1 blockade between intra-adrenal and subcutaneous tumors in both models. We assessed adrenal tumor growth by serial ultrasound while subcutaneous tumor growth was assessed by caliper-based measurements. Renca adrenal tumors showed no detectable growth delay following anti-PD-1 treatment compared with isotype control, whereas subcutaneous Renca tumors grew more slowly in the anti-PD-1 cohort and one in six regressed completely (**Figure 6C**). The differences in response between subcutaneous and adrenally-implanted tumors following PD-1 blockade were consistent with adrenal resistance to ICI in the Renca model.

However, due to Renca exhibiting variable responsiveness to PD-1 blockade in prior publications [Pham et al 2022, Fabian et al 2017, Kunimura et al 2020, O'Shaughnessy et al 2018], we next sought to validate these findings in an additional tumor line. Given that MC-38 is known to be highly responsive to anti-PD-1 blockade in vivo, we tested the same experimental design using subcutaneous and adrenally-implanted MC-38 tumors. [Juneja et al 2017, Homet Moreno et al 2016, Inoue et al 2025, Vikram et al 2017, Lecocq et al 2021]. Unlike the Renca model, tumor growth of MC-38 tumors was not significantly increased in the adrenal gland compared to the subcutaneous location following IgG treatment, although there was a trend toward faster growth in the adrenally-implanted tumors (p=0.059) (**Figure 6D**). Additionally, unlike the Renca model where complete tumor regression was rare in subcutaneous tumors and completely absent in adrenally implanted tumors, we observed complete regression of all MC-38 tumors in both subcutaneous (N=6/6) and adrenal (N=6/6) locations following treatment with PD-1 blockade (**Figure 6D**) Importantly, however, whereas the subcutaneous tumors never grew back after responding completely to PD-1 blockade, we observed delayed tumor regrowth of the adrenally-injected tumors after approximately 75 days (**Figure 6E**) with 2 out of 4 mice developing both recurrent tumors in the adrenal gland as well as diffusely within the peritoneal cavity consistent with a carcinomatosis pattern. Of note, we observed significantly improved survival in mice with subcutaneous tumors treated with PD-1 blockade in the Renca and MC-38 models. Evidence of adrenal resistance to PD-1 blockade was observed for both, but with different manifestations of resistance to its anti-tumor effects (**Figure 6E**).

*PD-1 blockade fails to expand the adrenal effector-memory response*

Next, we sought to analyze the role of PD-1 blockade on the Renca murine model to interrogate potential mechanisms of ICI resistance in the adrenal tumors. First, as shown in **Figure 7A** and similar to our human data (**Figure 1B, 2C, 3C, 4D, 4E**) we demonstrated higher frequency of PD-1 expression in T cells from non-tumor bearing murine adrenal glands compared to splenic T cells. In particular, the percent of CD8+ T cells that expressed PD-1 was significantly higher in murine adrenal glands compared to the spleen (44.0±16.9 vs 7.06±4.3, $p<0.05$). We then performed immune characterization of subcutaneous and adrenal Renca tumors on Day 15 post implantation and Day 12 post anti-PD-1 therapy (**Figure 7B**) at a time point where we observed no difference in tumor growth between adrenal tumors with and without PD-1 blockade compared to differences in tumor growth in the subcutaneous model following PD-1 blockade (**Figure 6C, 7C**). At the conclusion of the study, the anti-PD-1 treated cohort displayed an increase in PD-1 expression for the subcutaneous tumors compared to spleens on both CD4+ T cells (fold change 3.03±1.0 vs 0.51±0.06, $p<0.05$) and CD8+ T cells (fold change 4.4±1.6 vs 0.82±0.2, $p<0.05$), whereas there was no difference in PD-1 expression for the adrenal tumor groups (**Figure 7D**). Similarly, anti-PD-1 treatment resulted in an increase in CD69 expression on both CD4+ T cells (fold change 2.5± 0.3 vs 1.2±0.03, $p<0.05$) and CD8+ T cells (fold change

2.5±0.3 vs 1.0±-0.1, $p<0.05$) in subcutaneous Renca tumors, which was not observed on the tumor-infiltrating CD4+ and CD8+ T cells in the adrenal tumor mice (**Figure 7E**). Likewise, there was increased co-expression of both PD-1 and CD69 following PD-1 blockade on both CD4+ T cells (fold change 2.9±0.4 vs 0.6±0.1, $p<0.05$) and CD8+ T cells (fold change 9.6±1.7 vs 0.4±0.1, $p<0.05$) in mice with subcutaneous tumors, whereas there was no difference in these subsets in the adrenal tumor groups (**Figure 7F**). To expand upon this analysis, we then analyzed the effect that PD-1 blockade had on T cell subsets in the Renca murine model. We observed that in mice with subcutaneous tumors, there was a significant increase in CD4+ effector memory T cells when given anti-PD-1 treatment (fold change 2.3±0.4 vs 1.1±0.1, $p<0.05$), while a significant decrease in CD4+ effector memory T cells was seen in the adrenal tumor groups (fold change 0.7±0.1 vs 1.2±0.01 $p<0.05$) (**Figure 7G**). Similarly, there was an increase in CD8+ effector memory T cells in mice with subcutaneous tumors following anti-PD-1 treatment (fold change 2.7±0.2 vs 1.2±0.2, $p<0.05$), which was not observed in the mice with adrenally implanted tumors (**Figure 7H**). Due to the lack of effector memory T cell increase in the adrenal tumor groups treated with anti-PD-1, we aimed to further elucidate potential mechanisms of adrenal ICI resistance through single-cell RNA sequencing analysis to assess TCR clonotype predominance.

*Adrenal T cells exhibit reduced TCR diversity, and shared clonotypes undergo tissue-associated phenotypic remodeling*

To determine whether the compartment-specific transcriptional states observed in adrenal T cells were associated with differences in clonal architecture and could provide insight into adrenal PD-1 resistance, we integrated productive paired alpha-beta TCR sequences with single-cell transcriptional-state annotations. Across 9,794 TCR-bearing cells, clone-resolved visualization revealed marked inter-donor heterogeneity but recurrent clonal expansion in adrenal tissue (**Figure 8A**). Donors 1, 3, and 4 contained prominent adrenal-expanded clonotypes associated with activation and/or effector-memory states, whereas matched PBMC repertoires were dominated by smaller naïve/central memory-associated clonotypes. Donor 2 was an important exception, with a highly expanded cytotoxic PBMC clonotype comprising 23.5% of circulating TCR-bearing cells (**Figure 8A**).

To compare repertoire diversity independently of TCR-cell recovery, we rarefied each donor's paired adrenal and PBMC repertoires to the smaller TCR-bearing cell count before calculating Hill diversity. Adrenal repertoires showed reduced clonotype diversity in donors 1, 3, and 4, whereas donor 2 displayed the opposite pattern (**Figure 8B**). Consistent with these findings, the fraction of cells belonging to expanded clonotypes was greater in adrenal tissue for donors 1, 3, and 4 but lower in donor 2 (**Figure 8C,D)**. Thus, the dominant pattern in three of four donors was an adrenal repertoire with reduced diversity and greater representation of expanded clonotypes, superimposed on substantial inter-individual heterogeneity.

We next asked whether clonotypes present in both PBMC and adrenal samples contributed disproportionately to cross-compartment repertoire overlap. Cell-weighted overlap exceeded overlap based only on unique clonotype identities for every donor, both across all T cells and within memory/effector populations (**Figure 8E)**. Across all donors, the mean cell-weighted overlap was 26.5% for all T cells and 30.2% for memory/effector T cells, compared with 4.1% and 5.3%, respectively, when each shared clonotype was counted only once. These findings suggest cross-tissue repertoire overlap was concentrated within a relatively small number of expanded shared clonotypes rather than reflecting broad sharing of the overall TCR repertoire. Importantly, shared clonotypes occupied different transcriptional states in PBMC and adrenal samples. Among 230 clonotypes detected in both compartments within the same donor, 43.3-66.7% per donor differed in their dominant transcriptional state between PBMC and adrenal samples (**Figure 8F**). These data provide direct within-clonotype evidence that local tissue context is associated with T cell state remodeling.

To further examine whether tissue-associated transcriptional remodeling was driving the observed divergent transcriptional profiles between PBMC and adrenal T cells, and to explore whether differences in the transcriptional profiles between circulating versus adrenal antigen-specific T cells could help explain the adrenal resident T cell PD-1 blockade resistance, we compared the TCR sequence similarity between the productive TCRs identified in our studies to those in curated TCR databases. Database matching identified 212 cells carrying TCRs assigned to putative CMV-, EBV-, or influenza-associated categories, comprising 124 CMV candidates, 21 EBV candidates, and 67 influenza candidates (**Supplemental Figure 4A, B**). Of these, 73 CMV-, 15 EBV-, and 13 influenza-associated candidate T cells were detected in adrenal tissue. Within each putative viral category, activation-associated states comprised a larger fraction of the adrenal than PBMC T cells (**Supplemental Figure 4C**), and clone-size distributions were skewed toward larger candidate CMV and EBV clones in adrenal tissues (**Supplemental Figure 4D,E**). Putative virus-associated clonotypes detected in both compartments frequently occupied different dominant states in blood and adrenal tissue (**Supplemental Figure 4F**). These observations are consistent with tissue-associated remodeling among clonotypes that resemble previously reported antiviral TCRs. Importantly, despite the relatively high frequency of putative antigen-specific T cells with an activated phenotype in the adrenal tissues, these cells did not exhibit an exhausted state. This suggests that the observed PD-1 blockade resistance observed in adrenal cancers may be partially due to either a lack of exhausted antigen-specific T cells that can be reinvigorated, or a disproportionate number of viral-specific T cells that occupy an adrenal niche.

## Discussion:

In this study, we provide comprehensive characterization of the adrenal immune microenvironment in both human samples and murine models to investigate the potential mechanisms of adrenal metastatic resistance to ICI. Our analysis of RCC patients with adrenal metastasis from both our institution and Caris Life Sciences' multi-center clinical and genomic CARIS database showed no survival benefit using ICIs, unlike RCC patients with extra-adrenal metastasiss, which supports studies from other institutions characterizing the adrenal gland as a sanctuary site for ICI. These data highlight the clinical relevance of adrenal resistance to ICI and underscore the need for further research into potential mechanisms. Historically, the adrenal glands have been considered an immune privileged site [Deng et al 2025, Bedogni et al 2020]. Immune privileged organs such as the eyes or gonads have been shown to tolerate exposure to antigens without mounting a normal inflammatory response, likely due to an evolutionary mechanism to protect these critical organs from autoimmunity [Stein-Streilein et al 2014, Arck et al 2008, Niederkorn et al 2006, Benhar et al 2012, Apps et al 2009]. The adrenal glands are similarly crucial to survival, as they are the center of steroidogenesis in the host. Adrenal insufficiency results in many deleterious and frequently life-threatening, effects including severe hypotension and eventual cardiovascular collapse. Therefore, adrenal resistance to ICI could be the byproduct of an evolutionarily protective mechanism. Immune privilege has traditionally been linked to a dearth of immune cells whereas our results have shown that surprisingly, the adrenal glands cannot be classified as immune deserts. They instead have a robust PD-1+ T cell infiltrate that appears to be connected to mechanisms of adrenal ICI resistance.

Paired single-cell profiling expanded upon this phenotype showing a reproducible activation- and tissue-conditioning program with increased *PDCD1*, *NR4A1*, *AREG*, *DUSP4*, immediate-early, heat-shock, hypoxia, TNF/NF-κB, and mTORC1-associated programs. These findings are consistent with an immune rich but qualitatively specialized tissue compartment rather than one with generalized T cell exclusion. Importantly, PD-1 expression and cytokine competence alone do not exclude exhaustion, and our data indicates that adrenal T cells are not uniformly hypofunctional and do not show a dominant transcriptional state of exhaustion.

The paired TCR sequencing data provide a second, independent layer of evidence supporting a pattern consistent with T cell lineage-dependent tissue conditioning and altered state composition, rather than a single global activation axis in the adrenal glands. The TCR clonotypes present in both blood and adrenal tissues frequently occupied different dominant transcriptional states. This observation suggests clonotype transcriptional profiles and activation states are at least partially linked to local tissue milieus. Whether this reflects local antigen encounter, cytokine exposure, metabolic adaptation, selective retention, or a combination of these processes remains unresolved. Curated-database matching further identified candidate CMV-, EBV-, and influenza-associated TCRs in adrenal tissue. Virus-specific or other cancer-unrelated bystander T cells are known to populate human tumors, making this observation biologically plausible. Our findings that adrenal antigen-specific T cells maintain an activated phenotype in the absence of exhaustion may shed light on potential mechanisms of ICI resistance unique to this tissue compartment. Although speculative, our data suggest the adrenal glands may harbor a disproportionate frequency of viral-specific T cells, which may limit the recruitment and/or prevent the expansion of cancer-specific T cells. Additionally, differences in the transcriptional profiles between clonotype matched T cells from the blood and adrenal glands may reflect inherent differences in how antigen-specific T cells function between these two compartments. Both of these mechanisms could contribute to the adrenal gland's role as a sanctuary site for metastatic growth that is ICI resistant. However, direct HLA-matched peptide-MHC validation and functional testing against viral and tumor antigens would be required to determine whether bystander clonotypes meaningfully compete with or alter tumor-reactive T cells responses in the adrenal niche and should be focused on in future studies.

In our retrospective and prospective human analyses, we consistently observed adrenal PD-1+ T cells in both the TME of adrenal metastases as well as within the microenvironment of the non-tumor bearing adrenal gland. While PD-1 expression can be a marker of early antigen-specific T cell activation, it is more commonly associated with T cell exhaustion, particularly in cancer patients [Chi et al 2024, Weiss et al 2024, Chen et al 2026, Simula et al 2022, Bengsch et al 2020, Balanca et al 2021]. Yet paradoxically, our results show that these adrenal T cells are not classically exhausted, as they have a high degree of PD-1 and CD69 co-expression, HLA-DR positivity, and do not display hypofunctional in cytokine secretion of IFNγ, TNFα, and IL-2 following short term stimulation. Gene ontology pathways confirmed an activated PD-1+ T cell phenotype, especially among CD8+ T cells. Moreover, we observed that the adrenal glands are especially enriched for PD-1+ effector memory T cells. Yet, the question remains as to why adrenal ICI resistance still occurs despite the adrenal tissue harboring a robust population of effector memory T cells.

Through our novel murine models of adrenal metastasis via ultrasound-guided intra-adrenal implantation, we recapitulated the clinical scenario of adrenal resistance to PD-1 blockade in two distinct murine models of orthotopic adrenal tumors. Moreover, this model allowed us to assess the effects of PD-1 blockade on T cell subtypes. We observed that within the subcutaneous microenvironment, the addition of anti-PD-1 blocking antibodies resulted in a notable enrichment of effector memory T cells. In contrast, our immune characterization also revealed that anti-PD-1 treatment failed to enrich the frequency of effector memory T cells in the adrenal glands. The lack of T cell effector memory reinvigoration in the adrenal gland could be the key towards uncovering how adrenal ICI resistance is maintained. The mechanisms behind this potential dysregulation of T cell effector memory cells are unclear, but could include epigenetic modifications, T cell exhaustion associated transcription factors, or persistent antigen stimulation [Ghoneim et al 2017, Wu et al 2026, Ahn et al 2024]. Moreover, inhibited T cell effector responses in the adrenal gland may be secondary to protective mechanisms to avoid autoimmunity or CD8+ T cell mediated tissue damage [Wooldridge et al 2012, Gupta et al 2024].

One important limitation of our work is that our human studies did not evaluate completely normal adrenal glands. Non-tumor adrenal tissue from patients undergoing elective adrenalectomy for benign lesions was the closest approximation of the normal human adrenal gland that was available to us, we cannot fully rule out that the presence of a benign lesion (such as an adrenal adenoma) might introduce confounding effects on the adrenal immune microenvironment. Moreover, several of our analyses rely on flow cytometry, which has the advantage of providing clear detection of immune markers through fluorescent antibodies, yet is still subject to potential bias through gating strategies and data interpretation [Brestoff et al 2022, Liu et al 2024]. Lastly, our novel murine models of adrenal metastasis relay on orthotopic tumor implantation, which does not replicate the pathogenesis of adrenal metastasis in human patients in terms of naturally occurring tumor development [Stribbling et al 2024, Oliveira et al 2020].

Despite these limitations, our study surprisingly demonstrates that the adrenal gland is enriched for activated PD-1+ effector T cells in both human and murine adrenal glands. We further recapitulate the phenomenon of adrenal ICI resistance in two novel murine models of orthotopic adrenal tumors. Moreover, we have observed a diminished effector T cell response in murine adrenal tumors following treatment with PD-1 blockade. Our data also reveal that adrenal T cells exhibit an immunodominance of non-cancer detecting clonotypes, which could be the reason behind diminished T cell responses to PD-1 blockade. Ultimately, our findings highlight the growing field of research into adrenal metastasis. Given the poor outcomes in cancer patients with adrenal metastatic lesions, our data may help to direct future studies into reversing ICI resistance and advancing the development of novel therapeutic strategies for this under researched patient population.

## Materials and Methods:

### Mice:

Female BALB/c and C57BL/6 mice were procured from Jackson Laboratory. Unless otherwise noted, all mice were 8 – 12 weeks at the time of experimentation. All mice were housed in an American Association for Accreditation of Laboratory Animal Care approved, specific pathogen free facility. Animal studies were approved by the UC Davis Institutional Animal Care and Use Committee Protocol: 23537.

### In Vivo Murine Tumor Studies:

Mouse tumor cells lines for Renca and MC-38 were utilized for in vivo experimentation. All tumor cells were thawed and cultured in complete RPMI media supplemented with 10% fetal bovine serum (Genesee Scientific, NC1718120) and 1% penicillin-streptomycin

(Gibco, 15140122). Cells were incubated at 37°C in a humidified atmosphere with 5% $CO_2$. Culture media was changed every 2–3 days. Cells were passaged 3 times prior to injection. 1.5-2.0x10^6 tumor cells were suspended in 20 ul of collagen hydrogel (Advanced BioMatrix, 5074) for injection with a 28G needle with a 12.7 mm length tip (Embecta, 329461). Subcutaneous tumors were injected underneath the skin at the right flank as previously described. Subcutaneous tumor size was measured using calipers. Adrenal tumors were injected under direct visualization with a Vevo F2 ultrasound (Fujifilm VisualSonics, Inc., Toronto, Canada) equipped with a 128-element linear array transducer operating at 25 – 57 MHz frequency. Ultrasound images were reconstructed and analyzed in Vevo LAB software (v5.10.0). Mice were first anesthetized with 3-5% isoflurane (VetOne, 13985-528-60) on induction, then maintained on 1-2% isoflurane for the injection. Hair on the left upper flank was locally removed, and ultrasound gel (TENSpros, 50-5601-1) was applied. Using ultrasound visualization, the left adrenal gland was then identified and injected with tumor cells. Mice were followed for survival and tumor growth, measured using weekly ultrasound surveillance. Terminal endpoints were used per IACUC guidelines.

In Vivo Murine PD-1 Blockade Treatment:

Mice bearing subcutaneous or adrenal tumors received either anti-PD-1 antibody (BioCell, clone 29F.1A12, BE0273) or rat IgGa isotype control (BioCell, clone 2A3, BE0089) via intraperitoneal injections. Beginning on day 3 after tumor implantation, an initial loading dose of 500 µg in 100 µL was administered, followed by a biweekly maintenance dose of 200 µg in 100 µL of PBS over three weeks.

In Vivo Murine Tissue Processing:

Mice were euthanized at indicated time points. After euthanasia, organs were collected and processed into single cell suspension. Spleen, tumors, and adrenal glands were first mechanically processed. Adrenal glands were then further enzymatically processed in 0.1 mg/ml DNase (Worthington, LS002139) 1mg/ml Collagenase solution (Worthington, LS004188) for 30-40 min at 37 C on a shaker. All samples were then filtered and treated with RBC lysis buffer for 5 minutes on ice, washed, and then suspended in PBS for subsequent flow cytometry analysis.

In Vivo Murine Flow Cytometry:

Mouse samples were prepared into single cell suspension in PBS. Cells were then incubated with Fc block (Biolegend, 101320), then stained with fluorochrome-conjugated antibodies. Antibodies utilized include the following: CD3 BV785 (Biolegend, clone 17A2, 100231), CD4 BV605 (Biolegend, clone RM4-5, 100548), CD8 APC (Biolegend, clone 53-6.7, 100712), CD45 BUV395 (BD Biosciences, clone 30-F11, 564279), CD69 FITC (BD Biosciences, clone H1.2F3, 553238), PD-1 PE-Cy7 (Biolegend, clone 29F.1A12, 135216), CD62L PerCP-Cy5.5 (Biolegend, clone MEL-14, 104432), CD44 PE (Biolegend, clone IM7, 103008). Live/dead staining was performed using eBioscience™ Fixable Viability Dye eFluor™ 780 (Invitrogen, 65-0865-14).

All mouse cells were analyzed using a BD Fortessa flow cytometer (BD) and results were analyzed with FlowJo software (BD). Mouse T cells were gated for FACS using the following strategies: singlets, live, CD45+, lymphocytes, CD3+, CD4+ or CD8+.

Caris Life Sciences CODEai$^{TM}$ Data:

A retrospective analysis was conducted on tumor samples submitted by clinical physicians for molecular profiling at Caris Life Sciences (Phoenix, Arizona, USA), a College of American Pathologists (CAP)/Clinical Laboratory Improvement Amendments (CLIA)-certified laboratory. Hematoxylin and Eosin (H&E) stained formalin-fixed, paraffin-embedded (FFPE) slides of the patient's tumor underwent review by a board-certified pathologist or trained pathologist assistant. Tumor enrichment was achieved by harvesting targeted tissue using manual microdissection techniques. For whole transcriptome sequencing (WTS), a minimum of 10% tumor nuclei in the area of microdissection was required, with a minimum 10 mm 2 dissection area. Real-world overall survival (OS) information was obtained from insurance claims data. OS was calculated from time of tissue collection until date of death or last contact. Patients without contact/claims data for a period of at least 100 days were presumed deceased. Patients with documented clinical activity within 100 days prior to the latest data update were censored in the analysis. Kaplan-Meier estimates and log-rank p-values were calculated for molecularly defined patient cohorts using the python package lifelines (v0.30.0), where significance was determined as $P<0.05$. The deidentified sequencing data are owned by Caris Life Sciences and researchers can apply for data access by submitting a letter of intent.

Human Samples:

Fresh human blood, non-tumor-bearing adrenal tissue, and tumor samples were collected from patients who provided written informed consent for IRB-approved protocols: 218204, 2217090, and 2215078.

Immunohistochemistry:

Immunohistochemical staining (IHC) was performed on formalin-fixed, paraffin-embedded (FFPE) tissue microarrays (TMA). TMAs were stained for PD-1 (Abcam, clone EPR4877, ab137132). Slides were analyzed in a blinded fashion. H-score was determined using open-source QuPath Software.

Human Tissue Processing:

Whole blood was processed into PBMCs and plasma as previously described [Judge et al 2020]. Patient adrenal tissue and tumor specimens were collected from surgical specimens in RPMI at 4°C and processed into single cell suspension. First, all adrenal tissue and tumor specimens were washed, and any extraneous perinephric fat was removed. The samples were then mechanically processed. Adrenal tissue was then further enzymatically processed in 0.1 mg/ml DNase (Worthington, LS002139) 1mg/ml Collagenase solution

(Worthington, LS004188) for 30-40 min at 37 C on a shaker. All samples were then filtered and treated with RBC lysis buffer for 5 minutes, washed, and then suspended in PBS for subsequent flow cytometry or single cell sequencing analysis.

Ex Vivo Human Cytokine Stimulation:

Human cells in single cell suspension were cultured in RF10C and stimulated with 1x PMA/IO (Invitrogen:00497093) for 4 hours. After 1 hour, Golgi plug (BD Biosciences: 51-2301KZ) and Golgi stop (BD Biosciences: 51-2092KZ) were added. Cells were then stained for flow cytometry as described below.

Ex Vivo Human Flow Cytometry:

Using single cell suspensions, cells were incubated with Human TruStain Fc Receptor Blocking Solution (BioLegend, 422302) and then stained with fluorochrome-conjugated antibodies. Antibodies utilized include the following: CD3 FITC (Biolegend, clone HIT3a, 300744), CD4 AF700 (Invitrogen, clone RPA-T4, 56-0048-42), CD8 BV785 (Biolegend, clone RPA-T8, 301046), CD45 BV510 (Biolegend, clone H130, 304036), CD45 PE-Texas Red (Biolegend, clone H130, MHCD4587), PD-1 BV711 (Biolegend, clone EH12.2H7, 329428), PD-1 PE-Cy7 (Biolegend, clone A17188B, 621616), HLA-DR Pacific Blue (Biolegend, clone L243, 307633), CCR7 PE-Cy7 (BD Biosciences, clone 3D12, 758102), CD45ra APC (Biolegend, clone HI100, 983004), CD69 BV650 (BioLegend, clone FN50, 310934), CD103 PE (Biolegend, clone Ber-ACT8, 350206), IFNg APC (BioLegend, clone 45.B3, 502512), TNFa BV650 (BioLegend, clone MAb11, 502938), IL-2 PerCp-Cy5.5 (BioLegend, clone MQ1-17H12, 500322). Live/dead staining was performed using eBioscience™ Fixable Viability Dye eFluor™ 780 (Invitrogen, 65-0865-14). All human cells were analyzed using a BD Fortessa flow cytometer (BD) and results were analyzed with FlowJo software (BD). Human T cells were gated for FACS using the following gating strategies: singlets, live, CD45+, lymphocytes, CD3+, CD4+ or CD8+.

Single-cell sample preparation and 10x Genomics gene-expression/TCR library generation

For the sequencing cohort, cryopreserved cells were thawed in prewarmed RF10 medium comprising RPMI 1640 without glutamine, 10% Nu-Serum, 2 mM L-glutamine, 1x non-essential amino acids, 1 mM sodium pyruvate, 1x penicillin-streptomycin, and 15 mM HEPES. When cell yield permitted, adrenal suspensions underwent live-cell enrichment using the EasySep Dead Cell Removal (Annexin V) Kit (STEMCELL Technologies, 17899), and PBMC and adrenal samples underwent CD3-positive enrichment using the EasySep Release Human CD3 Positive Selection Kit (STEMCELL Technologies, 17751), according to the manufacturer's instructions. Low-yield adrenal samples were loaded without one or both enrichment steps. Aliquots collected after thawing and, where applicable, after live-cell or CD3-positive enrichment were stained with Erythrosin B and counted using a CellDrop FLi Automated Cell Counter (DeNovix). Processed cells were immediately advanced to single-cell library preparation.

Cells were resuspended at approximately 2,200 cells/microliter and processed on the 10x Genomics Chromium X platform using the Chromium GEM-X Universal 5-prime Gene Expression v3 4-plex on-chip multiplexing workflow (CG000770); all available cells were loaded for the low-yield adrenal samples ADO272 and ADO284. Gene-expression and V(D)J TCR libraries were prepared according to the manufacturer's modified on-chip multiplexing protocol. Libraries were quantified using a Qubit 4 Fluorometer (Thermo Fisher Scientific) with the High Sensitivity dsDNA Assay Kit (Invitrogen, Q33231), and library quality was assessed using a TapeStation 4200 (Agilent Technologies) with High Sensitivity D5000 ScreenTape and reagents (5067-5592 and 5067-5593). Libraries were sequenced by Novogene (Sacramento, California) on a NovaSeq X Plus using a 25B flow cell and paired-end 150-bp reads. Downstream analysis began from Cell Ranger filtered feature-barcode matrices and filtered V(D)J contig-annotation files.

Gene-expression quality control, integration, and annotation

Matrices from four paired donors (eight samples) were imported into Seurat version 5.4.0 [Hao et al 2021]. Genes detected in fewer than three cells were removed. Cells were retained when they contained 200-4,000 detected features and no more than 20% mitochondrial transcripts. Counts were normalized using SCTransform while regressing percent mitochondrial reads. Principal-component analysis was followed by Harmony version 1.2.4 integration using donor identity as the grouping variable [Korsunsky et al 2019], preserving tissue as the biological comparison. UMAP and nearest-neighbor graphs were calculated from the first 30 Harmony dimensions, and global T-cell clusters were identified at resolution 0.8.

T cells were selected using concordant T-lineage module identity and canonical genes including CD3D, CD3E, TRAC, and CD247. CD4 identity used CD4, IL7R, CCR7, LTB, MAL, and ICOS; CD8 identity used CD8A, CD8B, NKG7, CCL5, GZMK, and PRF1. Biological states were assigned by integrating cluster-enriched genes with curated marker modules for naive, central-memory, effector-memory, cytotoxic, tissue-resident memory, follicular-helper, regulatory, MAIT, exhausted, proliferating, interferon-response, and activation programs. CD4 and CD8 cells were then reclustered independently after normalization, selection of 2,000 variable genes, exclusion of TCR genes from variable features, scaling with regression of mitochondrial percentage, PCA, donor-based Harmony integration, UMAP, and clustering at resolution 0.7.

Donor-level composition, pseudobulk differential expression, and pathway analysis

Cell state fractions and module scores were calculated within donor and tissue, preserving donor as the unit of replication. Paired effects are reported as adrenal-minus-PBMC differences. Because the cohort included four paired donors, compositional and module-score panels emphasize effect size and directional concordance rather than asymptotic cell-level p-values. For differential expression, raw counts were aggregated within each donor-tissue combination for global, CD4-like, and CD8-like analyses. Genes were filtered with edgeR version 4.6.3 filterByExpr (minimum count 5), normalized by the trimmed mean of M values, and analyzed with robust quasi-likelihood negative-binomial models using design ~ donor + tissue. Adrenal was the positive coefficient. Differential expression required Benjamini-Hochberg FDR < 0.05 and absolute log2 fold change >= 0.5. Donor aggregation avoids treating cells from the same participant as independent replicates [Squair et al 2021].

Genes were ranked as sign(log2 fold change) multiplied by -log10 nominal p value. Preranked enrichment was performed with fgseaMultilevel (fgsea version 1.34.2) using Hallmark, Reactome, and Gene Ontology Biological Process gene sets obtained through msigdbr version 26.1.0. Gene sets containing 15-500 genes were tested, and FDR was controlled by the Benjamini-Hochberg method. Non-redundant, biologically interpretable terms were prespecified for display; full results are supplied as source-data tables.

Lineage-resolved GSEA and KEGG scoring

CD4 and CD8 cells were analyzed separately by the same donor-blocked pseudobulk and preranked GSEA procedures used for the global T-cell contrast. For complementary KEGG visualization, raw counts were normalized to 10,000 transcripts per cell and log1p transformed. Mean expression was calculated for nine prespecified KEGG Legacy gene sets: T-cell receptor signaling, cytokine-cytokine receptor interaction, mTOR signaling, apoptosis, antigen processing and presentation, natural-killer-cell-mediated cytotoxicity, glycolysis/gluconeogenesis, oxidative phosphorylation, and ribosome. Scores were averaged within lineage, donor, and tissue, and paired effects were calculated as adrenal minus PBMC. Donor-level points and median paired effects are displayed; no cell-level significance test was used for these four-donor pathway scores.

TCR processing and clonotype definition

V(D)J contigs were restricted to 10x-called cells that were productive, high confidence, full length, assigned to TRA or TRB, and contained nonempty nucleotide and amino-acid CDR3 sequences. When multiple chains were present, the highest-ranked TRB and TRA contigs were selected by UMI and read support. Productive alpha-beta T cells were required to contain exactly one TRB and one or two TRA chains. Clonotypes were defined within donor by paired TRA/TRB nucleotide CDR3 sequences, preventing identical strings from unrelated donors from being treated as one biological clone.

TCR diversity, overlap, and phenotype transitions

To control for unequal TCR sampling, PBMC and adrenal clone-size vectors were rarefied within donor to the smaller paired TCR-bearing cell count using 400 without-replacement iterations. Hill diversity was calculated at $q = 0$ (richness), $q = 1$ (exponential Shannon), and $q = 2$ (inverse Simpson). Normalized clonality was defined as 1 - Shannon entropy/log(observed clonotypes). Expanded-clone occupancy was the fraction of cells in clonotypes represented by at least two cells; largest-clone occupancy was the fraction in the most abundant clonotype. Unique-clonotype overlap was calculated from shared clonotypes relative to the union, and cell-weighted overlap was the fraction of cells belonging to shared clonotypes. A clonotype was shared when its donorqualified paired TRA/TRB definition occurred in both tissues. For shared-clone transitions, each clonotype was assigned its most frequent state within PBMC and adrenal; states were condensed to naive/TCM, TEM, cytotoxic, activation, TRM, and other. Sankey ribbon widths report the number of shared clonotypes following each transition.

Putative viral-associated TCR matches

Exact CDR3 sequence annotation records generated by the upstream IEDB, McPAS-TCR, VDJdb, and TCRdb matching pipeline were filtered to viral records. Disease and antigen fields were searched for CMV, EBV, influenza, YFV, and HIV terminology; YFV and HIV categories were excluded from display because of low abundance. Full paired-sequence keys were matched first, followed by donor-constrained paired-alpha/beta or single-chain CDR3 amino-acid keys when available. Duplicate database evidence was collapsed at the cell-pathogen-record level. When a cell matched more than one pathogen category, a single primary category was assigned by database-record support, hit-row count, and a prespecified CMV/EBV/influenza tie-break order. Matched-cell count, tissue distribution, phenotype composition, clone-size occupancy, and shared-clone phenotype were then summarized. These calls were treated as putative because specificity was not confirmed by donor HLA restriction, peptide-MHC multimer binding, or functional response assays.

Statistical Analysis

We used Microsoft Excel and GraphPad Prism (version 11.0.0, GraphPad Software) for statistical analysis. We analyzed the differences between two groups using paired or unpaired Student's t-test for parametric data, and the Mann-Whitney test or Wilcoxon signed-rank test for nonparametric data. Kaplan-Meier survival analysis was performed using the log rank test.

## Figure Legends:

**Figure 1.** Retrospective cohort of RCC patients with matched primary tumors and adrenal metastases displays an adrenal sanctuary site with resistance to ICI.
**(A)** Representative tissue micro array histology with low PD-1+ IHC staining compared to high PD-1+ staining. **(B)** Quantification of PD-1+ IHC staining of matched primary tumor, adrenal metastasis, and non-tumor adrenal from retrospective RCC patient cohort (n=22). **(C)** Quantification of PD-1+ IHC staining stratified into two categories of patients treated with ICI prior to adrenal metastasectomy (n=5) and patients who are ICI naïve prior to adrenal metastasectomy (n=17). **(D)** Quantification of metastatic lesion tumor growth in RCC patients while on ICI treatment (n=5) based on adrenal location compared to extra-adrenal location (bone, lung, liver). **(E)** Representative CT scan images for a RCC patient treated with ICI showing of change in growth of adrenal metastasis compared to liver metastasis. **(F)** Kaplan Meier survival curve of RCC patients with adrenal metastasis (n=132) stratified into two groups: patients treated with ICI and patients who were not treated with ICI. **(G)** Kaplan Meier survival curve of RCC patients with extra-adrenal metastasis (bone, lung, liver) (n=1327) stratified into two groups:

patients treated with ICI and patients who were not treated with ICI. P values were determined with ANOVA (B), two tailed unpaired Student's T test (C, D), and Mann U Whitney test (F, G): *p<0.05 **p<0.01 ***P<0.001

**Figure 2.** Prospective cohort of renal cell carcinoma patients with adrenal metastasis demonstrates adrenal microenvironment with prevalent T cells displaying PD-1+ and CD69+ immunophenotype.
**(A)** Gross specimen (bisected) of RCC adrenal metastasis with corresponding CT scan image prior to surgery. **(B)** Quantification of T cells (n=4) with representative FACS plots. **(C)** Quantification of PD-1+ expression (n=3) with representative FACS plots. **(D)** Quantification of CD69+ expression (n=4) with representative FACS plots.
P values were determined with two tailed unpaired Student's T test (B, C, D) *p<0.05 **p<0.01 ***P<0.001

**Figure 3.** Prospective analysis of human non-tumor adrenal tissue and matched PBMCs from benign adrenalectomy patients displays adrenal microenvironment with prevalent PD-1+ T cells with activated phenotype
**(A)** Quantification of T cells per proportion of lymphocytes (n=17) versus all live CD45+ cells (n=21) **(B)** Quantification of CD4+ and CD8+ T cell subsets (n=25) with representative FACS plots. **(C)** Quantification of PD-1+ expression (n=25) with representative FACS plots. **(D)** Quantification of CD69+ expression (n=25) with representative FACS plots. **(E)** Quantification of PD-1+ CD69+ co-expression (n=25) with representative FACS plots. **(F)** Quantification of HLA+ expression (n=14). **(G)** Quantification of PD-1+ HLA-DR+ co-expression (n=12) **(H)** Quantification of IFNγ+ expression (n=5). **(I)** Quantification of TNFα+ expression (n=5) **(J)** Quantification of IL-2 expression (n=4).
P values were determined with two tailed paired Student's T test (A, B, C, D, E, F, G) and two tailed unpaired Student's T test (G, H, I) *p<0.05 **p<0.01 ***P<0.001

**Figure 4.** Prospective analysis of human non-tumor adrenal tissue and matched PBMCs displays increased effector memory and tissue resident T cell subsets.
**(A)** Quantification of CD4+ T cell subsets (n=6) with representative FACS plots. **(B)** Quantification of CD8+ T cell subsets (n=6) with representative FACS plots**.** **(C)** Quantification of CD69+ CD103+ tissue resident T cells (n=6) with representative FACS plots. **(D)** Quantification of PD-1+ CD4+ T cell subsets (n=6) with representative FACS plots. **(E)** Quantification of PD-1+ CD8+ T cell subsets (n=6) with representative FACS plots. **(F)** Quantification of PD-1+ tissue resident T cells (n=6). P values were determined with two tailed unpaired Student's T test (A, B, C, D, E, F) *p<0.05 **p<0.01 ***P<0.001

**Figure 5.** Paired single-cell profiling identifies an adrenal-associated T-cell activation and tissue-conditioning program.

**(A)** UMAP of 15,060 T cells from matched PBMC and non-tumor adrenal samples from four donors, annotated into 12 transcriptional states after donor-aware integration and reclustering. **(B)** Mean T cell state composition by tissue across the four paired donors. **(C)** Mean paired adrenal-minus-PBMC change in the abundance of each T cell state; positive values indicate enrichment in adrenal tissue and negative values indicate enrichment in PBMCs. **(D)** Selected genes differentially expressed between adrenal and PBMC T cells in donor-blocked pseudobulk analysis. Points show log2 fold change (adrenal/PBMC); genes meeting the prespecified FDR and effect-size thresholds are indicated in the analysis source data. **(E)** Volcano plot of donor-blocked pseudobulk differential expression between adrenal and PBMC T cells. **(F)** Representative nonredundant pathways from preranked gene-set enrichment analysis. Positive normalized enrichment scores indicate adrenal enrichment and negative scores indicate PBMC enrichment; point size reflects gene-set size and shading encodes -log10 FDR. Donor is the unit of replication for paired composition and pseudobulk analyses. See Methods and source-data tables for statistical definitions and complete enrichment results.

**Figure 6.** In vivo murine model of adrenal metastasis treated with PD-1 blockade recapitulates adrenal ICI resistance
**(A)** Schema of tumor injection and Anti-PD-1 treatment, representative of 2 experiments with (n=4-6) for each group. **(B)** Example of set-up used for adrenal tumor injection. Ultrasound image pre-injection displaying the injection needle (green arrow) the adrenal gland (red arrow) and the top of the kidney (blue arrow). Ultrasound images of adrenal tumors at endpoint prior to collection (yellow arrow). Pictures of adrenal tumors prior to removal during necropsy (yellow arrow). Pictures of adrenal tumors with measurement scale. **(C)** Individual and grouped tumor growth curves for BalbC mice injected with Renca. **(D)** Individual and grouped tumor growth curves for C57BL/6 mice injected with MC-38. **(E)** Kaplan Meier survival curve for BalbC mice injected with Renca. **(F)** Kaplan Meier survival curve for C57BL/6 mice injected with MC-38. P values were determined with unpaired Student's T test (C, D) Log-rank (Mantel-Cox) test (E, F) *p<0.05 **p<0.01 ***P<0.001

**Figure 7.** In vivo murine model of adrenal metastasis demonstrates diminished T cell response in adrenal tumors treated with PD-1 blockade.
**(A)** Quantification of PD-1 expression in pooled non-tumor mice adrenal glands **(B)** Schema of tumor injection and Anti-PD-1 treatment representative of 1 experiment (n=3 per group). **(C)** Representative pictures of adrenal and subcutaneous tumors at necropsy. **(D)** Quantification of PD-1+ expression fold change in subcutaneous and adrenal tumor groups. **(E)** Quantification of CD69 expression fold change in subcutaneous and adrenal tumor groups. **(F)** Quantification of PD-1+ and CD69+ coexpression in subcutaneous and adrenal tumor groups with representative FACS plots. **(G)** Quantification of CD4+ T cell subsets in subcutaneous and adrenal tumor groups with representative FACS plots. **(H)** Quantification of CD8+ T cell subsets in subcutaneous and adrenal groups with representative FACS plots.

P values were determined with unpaired Student's T test (A, D, E, F, G, H) *p<0.05 **p<0.01 ***P<0.001

**Figure 8.** Adrenal TCR repertoires are clonally concentrated, and shared clonotypes undergo tissue-associated state remodeling.
(A) Clone-resolved honeycomb plots for matched PBMC and adrenal T cells from each donor. Circle area is proportional to clonotype size and color denotes the dominant transcriptional state; total TCR-bearing cells and observed clonotypes are shown for each sample. (B) Rarefied Hill diversity ($q = 0$, richness; $q = 1$, exponential Shannon; $q = 2$, inverse Simpson) for paired PBMC and adrenal repertoires. Within each donor, repertoires were repeatedly rarefied to the smaller paired TCR-bearing cell count before diversity estimation. (C) Donor-paired measures of clonal concentration, including normalized clonality, the fraction of cells in expanded clonotypes (at least two cells), and largest-clone occupancy. (D) Distribution of repertoire occupancy across clonotype-size bins for each donor and tissue. (E) Cross-tissue overlap calculated either by unique clonotype identity or by the fraction of cells belonging to shared clonotypes, shown for all T cells and for memory/effector T cells. (F) Sankey diagrams showing dominant transcriptional-state transitions for clonotypes detected in both PBMC and adrenal tissue within each donor. Ribbon width denotes the number of shared clonotypes following each transition. Clonotypes were defined within donors by productive paired TRA/TRB CDR3 nucleotide sequences as described in Methods.

**Supplemental Figure 1.** Additional Data
(A) Quantification of CD4+ and CD8+ T cells subsets (n=4).
P values were determined with two tailed paired Student's T test (A) *p<0.05 **p<0.01 ***P<0.001

**Supplemental Figure 2.** Global T-cell reclustering and donor-resolved composition of matched PBMC and adrenal samples.
**(A)** UMAP of the reclustered T cell compartment showing the 20 transcriptional clusters used for state annotation. **(B)** The same UMAP split by tissue, illustrating PBMC and adrenal occupancy of the integrated landscape. **(C)** Heat map of cluster-enriched genes used to support cluster annotation. **(D)** Dot plot of canonical marker modules used to assign biological T-cell states. **(E)** T-cell state composition for each donor and tissue. **(F)** Donor-paired adrenal-minus-PBMC changes in T-cell-state abundance. Positive values indicate adrenal enrichment; negative values indicate PBMC enrichment. Individual donors are shown separately, allowing assessment of directional concordance across the four paired donors.

**Supplemental Figure 3.** Lineage-resolved single-cell analysis demonstrates adrenal-associated remodeling within both CD4 and CD8 T cells.

**(A)** UMAPs of independently reclustered CD4 and CD8 T cells annotated by biological state. **(B)** Lineage-specific UMAPs split by tissue. **(C)** Mean lineage-specific T-cell-state composition in PBMC and adrenal samples. **(D)** Expression distributions for selected activation/tissue-associated, naive-memory and cytotoxic genes within CD4 and CD8 lineages. **(E)** Selected lineage-specific donor-blocked pseudobulk differential-expression effects, displayed as log2 fold change (adrenal/PBMC). **(F)** Representative lineage-resolved pathways from preranked gene-set enrichment analysis. **(G)** Donor-paired adrenal-minus-PBMC effects for prespecified KEGG pathways in CD4 and CD8 T cells. Points denote individual donors; positive values indicate higher pathway scores in adrenal tissue and negative values indicate higher scores in PBMCs. Donor, rather than individual cells, is the unit of replication.

**Supplemental Figure 4.** Putative virus-associated TCR database matches show tissue-skewed distribution and transcriptional-state remodeling.
**(A)** T cells carrying TCR sequences with putative CMV-, EBV- or influenza-associated database matches projected onto the global T-cell UMAP; point shape denotes tissue and color denotes candidate pathogen category. **(B)** Distribution of candidate CMV-, EBV- and influenza-associated cells between PBMC and adrenal tissue. **(C)** Transcriptional-state composition of candidate virus-associated cells by tissue and pathogen category. **(D)** Clone-size distributions for candidate virus-associated repertoires. **(E)** Tissue distribution of the most abundant candidate clonotypes within each pathogen category. **(F)** Dominant transcriptional-state assignments in PBMC and adrenal tissue for candidate virus-associated clonotypes detected in both compartments.

**Funding and Acknowledgements:**
This research was funded in part by NIH grant 1T32CA251007 (NML, CJS). This research was also supported in part by funds from the UC Davis Comprehensive Cancer Center, the UC Davis Department of Surgery, and the UC Davis Flow Cytometry Shared Resource Laboratory, with funding from NCI P30 CA093373 (Comprehensive Cancer Center) and S10 RR 026825- BD "Fortessa" cytometer, with technical assistance from Bridget McLaughlin and Jonathan Van Dyke. Specimens were provided by the UC Davis Pathology Biorepository, which is jointly funded by the UCD Comprehensive Cancer Support Grant (CCSG) awarded by the NCI (P30 CA093373) and the UCD Department of Pathology and Laboratory Medicine. We thank and acknowledge Qian Chen for assistance with immunohistochemistry staining. De-identified sequencing data from Caris Life Sciences are owned by Caris Life Sciences. Researchers may apply for access to these data by contacting Andrew Elliott (aelliott@carisls.com) and submitting a letter of intent.

**Data Availability:** Publicly available data for this project can be accessed through figshare under DOI https://doi.org/10.6084/m9.figshare.33992782

**Author Contributions:** NL- conceptualization, methodology, investigation, data curation, validation, formal analysis, visualization, writing – original draft, writing – review & editing, funding acquisition; JU- investigation, data curation; CV- investigation, data curation; AT- investigation, data curation; JS- investigation, data curation, writing – review & editing; OO- investigation, data curation; CS- investigation, data curation; TZ- investigation, data curation; MK- investigation, data curation; ML- investigation, data curation, writing – review & editing; MP- investigation, data curation, writing – review and editing; AAM- investigation, data curation; TT- investigation, data curation; JX- writing – review & editing, methodology, resources; WKR- writing – review & editing, methodology, resources; WB- writing – review & editing, methodology, resources; JK- writing – review & editing, methodology, resources; SG- writing – review & editing, methodology, resources, KM- investigation, data curation, validation, visualization, methodology, resources; MTC- investigation, data curation, validation, visualization, methodology, resources; AMM- writing – review & editing, methodology, resources; CG- conceptualization, writing – review & editing, resources; WM- writing – review & editing, methodology, resources; SJ- supervision, formal analysis, writing – review & editing, methodology; MJC- conceptualization, writing – review & editing, resources; AZ- investigation, data curation, formal analysis, visualization, software, resources, writing – original draft, writing – review and editing; RC- project administration, supervision, funding acquisition, conceptualization, methodology, formal analysis, resources, writing – original draft, writing – review & editing.

**Ethics Declaration:** This study was approved by the UC Davis Institutional Review Board (protocol numbers 2478568-1, 2217090-3, 2215078-2, and 218204). Participants provided informed consent before participating in the study. All animal studies were approved by the UC Davis Institutional Animal Care and Use Committee under protocol number 23537.

**Completing Interest Declaration:** There are no competing interests.

## Table 1

**Retrospective Cohort of Renal Cell Carcinoma Patients with Adrenal Metastasis, 2016-2024**

| Characteristic | | Number (N=22) | % |
|---|---|---|---|
| Age at Diagnosis | Average ± SD | 61 ± 10 | |
| Gender | Male | 21 | 95 |
| | Female | 1 | 5 |
| Initial Tumor Stage | Stage I | 5 | 23 |
| | Stage II | 0 | 0 |
| | Stage III | 10 | 45 |
| | Stage IV | 6 | 27 |
| | Unknown | 1 | 5 |
| Location of Metastasis | Isolated Adrenal Metastasis | 8 | 36 |
| | Extra-Adrenal Metastasis | 14 | 64 |
| Years to Adrenal Metastasis | Average ± SD | 4 ± 6 | |
| Immunotherapy | Pre-Adrenalectomy ICI | 5 | 23 |
| | No Pre-Adrenalectomy ICI | 17 | 77 |

## Table 2

**Prospective Cohort of Renal Cell Carcinoma Patients with Adrenal Metastasis, 2024-2026**

| Characteristic | | Number (N=4) | % |
|---|---|---|---|
| Age at Diagnosis | Average ± SD | 62 ± 9 | |
| Gender | Male | 4 | 100 |
| | Female | 0 | 0 |
| Initial Tumor Stage | Stage I | 0 | 23 |
| | Stage II | 1 | 25 |
| | Stage III | 2 | 50 |
| | Stage IV | 1 | 25 |
| | Unknown | 0 | 5 |
| Location of Metastasis | Isolated Adrenal Metastasis | 2 | 50 |
| | Extra-Adrenal Metastasis | 2 | 50 |
| Years to Adrenal Metastasis | Average ± SD | 4 ± 4 | |
| Immunotherapy | Pre-Adrenalectomy ICI | 1 | 25 |
| | No Pre-Adrenalectomy ICI | 3 | 75 |

## Supplemental Table 1

**Prospective Cohort of Elective Adrenalectomy Patients with Benign incidentalomas, 2024-2026**

| Characteristic | | Number (N=25) | % |
|---|---|---|---|
| Age at Diagnosis | Average ± SD | 57 ± 14 | |
| Gender | Male | 7 | 28 |
| | Female | 18 | 72 |
| Incidentaloma Dx | Adenoma | 18 | 72 |
| | Pheochromocytoma | 4 | 16 |
| | Other | 3 | 12 |

## Supplemental Table 2

**Prospective Cohort of Elective Adrenalectomy Patients for TCR Sequencing, 2025**

| Characteristic | | Number (N=4) | % |
|---|---|---|---|
| Age at Diagnosis | Average ± SD | 65 ± 5 | |
| Gender | Male | 2 | 50 |
| | Female | 2 | 50 |
| Incidentaloma Dx | Adenoma | 4 | 100 |

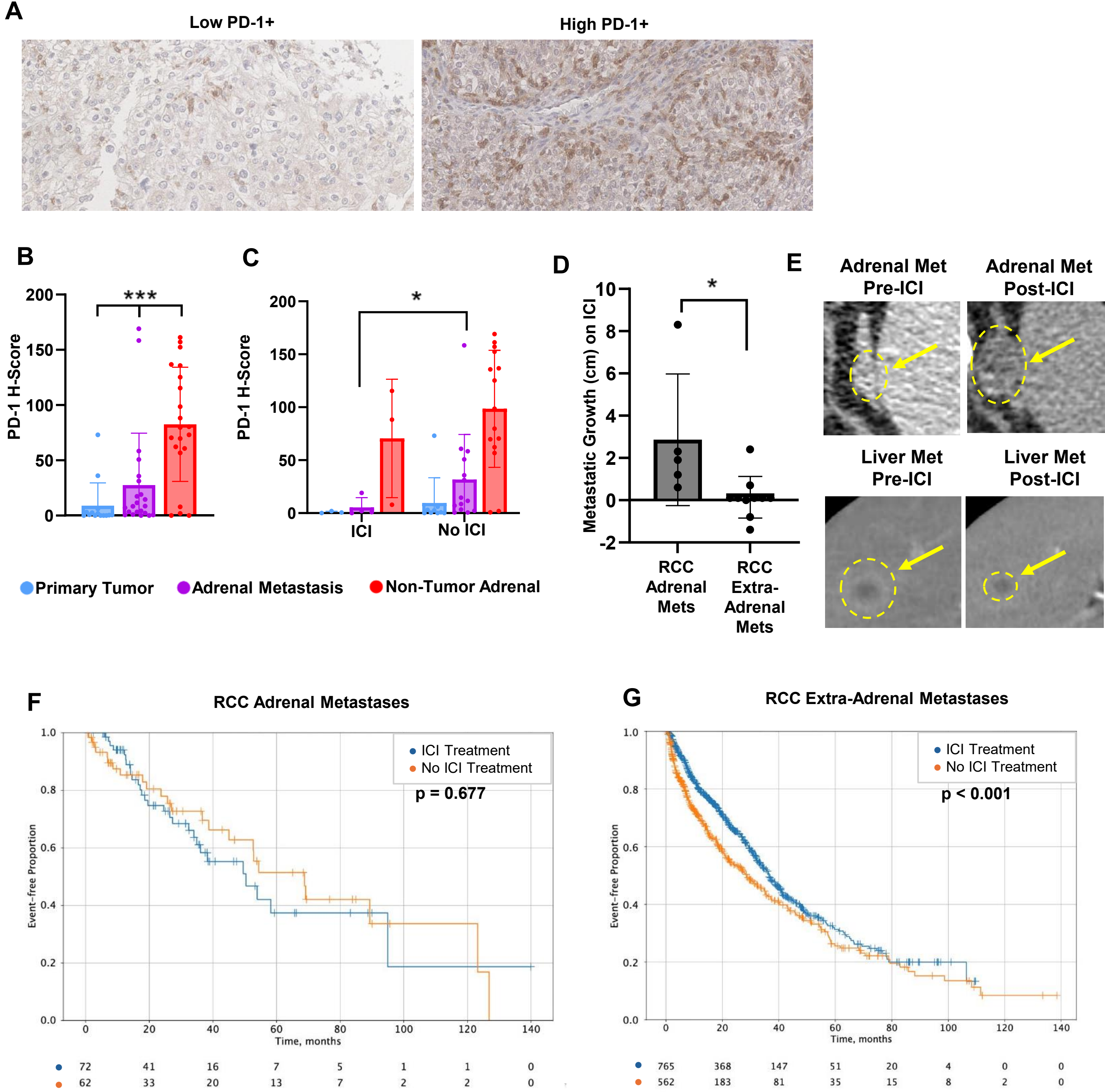

A
Low PD-1+
High PD-1+
B
PD-1 H-Score
200
150
100
50
0
***
C
PD-1 H-Score
*
ICI
No ICI
Primary Tumor
Adrenal Metastasis
Non-Tumor Adrenal
D
Metastatic Growth (cm) on ICI
10
8
6
4
2
0
-2
*
RCC Adrenal Mets
RCC Extra-Adrenal Mets
E
Adrenal Met Pre-ICI
Adrenal Met Post-ICI
Liver Met Pre-ICI
Liver Met Post-ICI
F
RCC Adrenal Metastases
ICI Treatment
No ICI Treatment
p = 0.677
Event-free Proportion
Time, months
72 41 16 7 5 1 1 0
62 33 20 13 7 2 2 0
G
RCC Extra-Adrenal Metastases
ICI Treatment
No ICI Treatment
p < 0.001
Event-free Proportion
Time, months
765 368 147 51 20 4 0 0
562 183 81 35 15 8 2 0

Figure 1

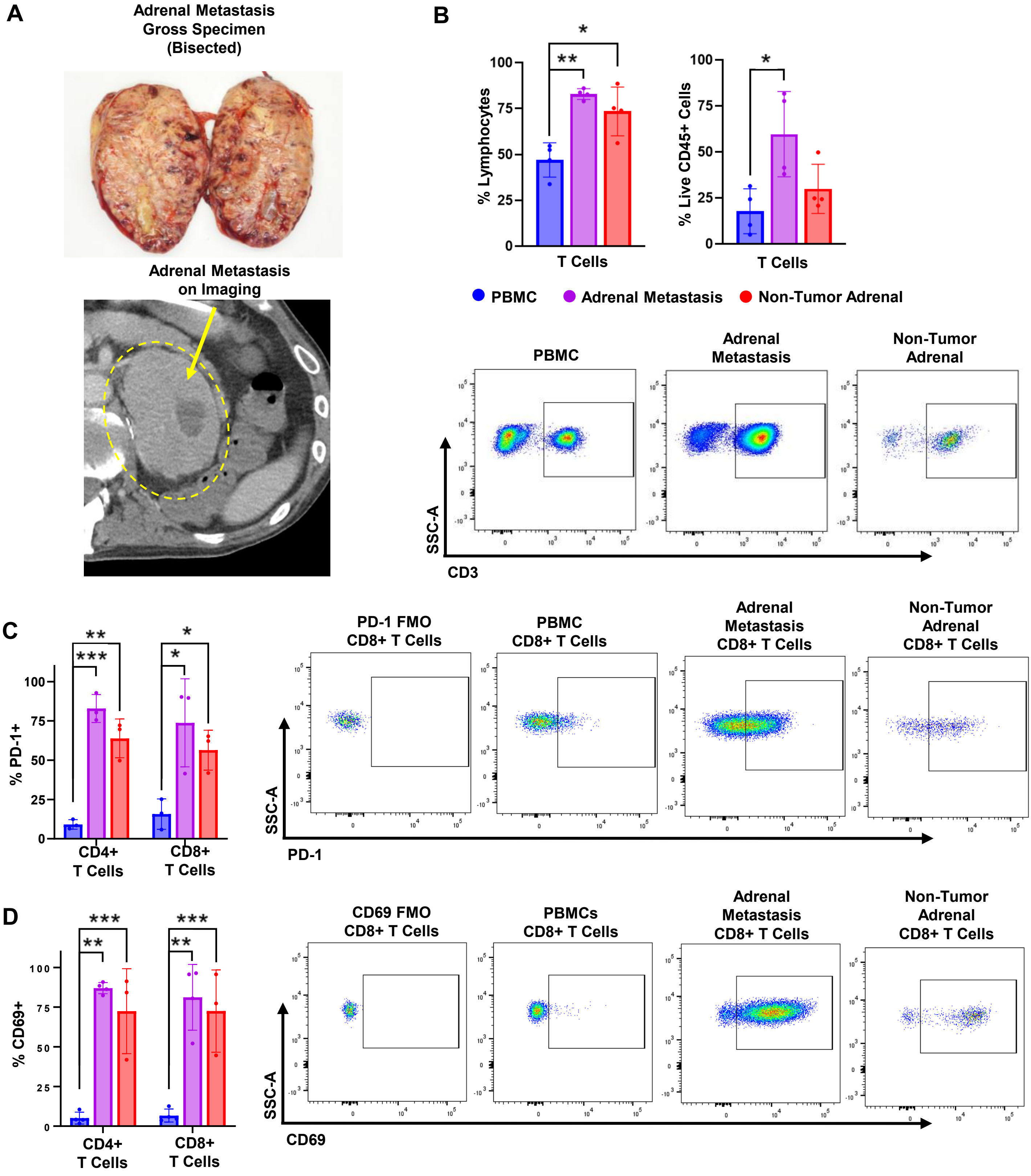

A
Adrenal Metastasis
Gross Specimen
(Bisected)
Adrenal Metastasis
on Imaging
B
% Lymphocytes
T Cells
% Live CD45+ Cells
T Cells
PBMC
Adrenal Metastasis
Non-Tumor Adrenal
PBMC
Adrenal
Metastasis
Non-Tumor
Adrenal
SSC-A
CD3
C
% PD-1+
CD4+
T Cells
CD8+
T Cells
PD-1 FMO
CD8+ T Cells
PBMC
CD8+ T Cells
Adrenal
Metastasis
CD8+ T Cells
Non-Tumor
Adrenal
CD8+ T Cells
SSC-A
PD-1
D
% CD69+
CD4+
T Cells
CD8+
T Cells
CD69 FMO
CD8+ T Cells
PBMCs
CD8+ T Cells
Adrenal
Metastasis
CD8+ T Cells
Non-Tumor
Adrenal
CD8+ T Cells
SSC-A
CD69

Figure 2

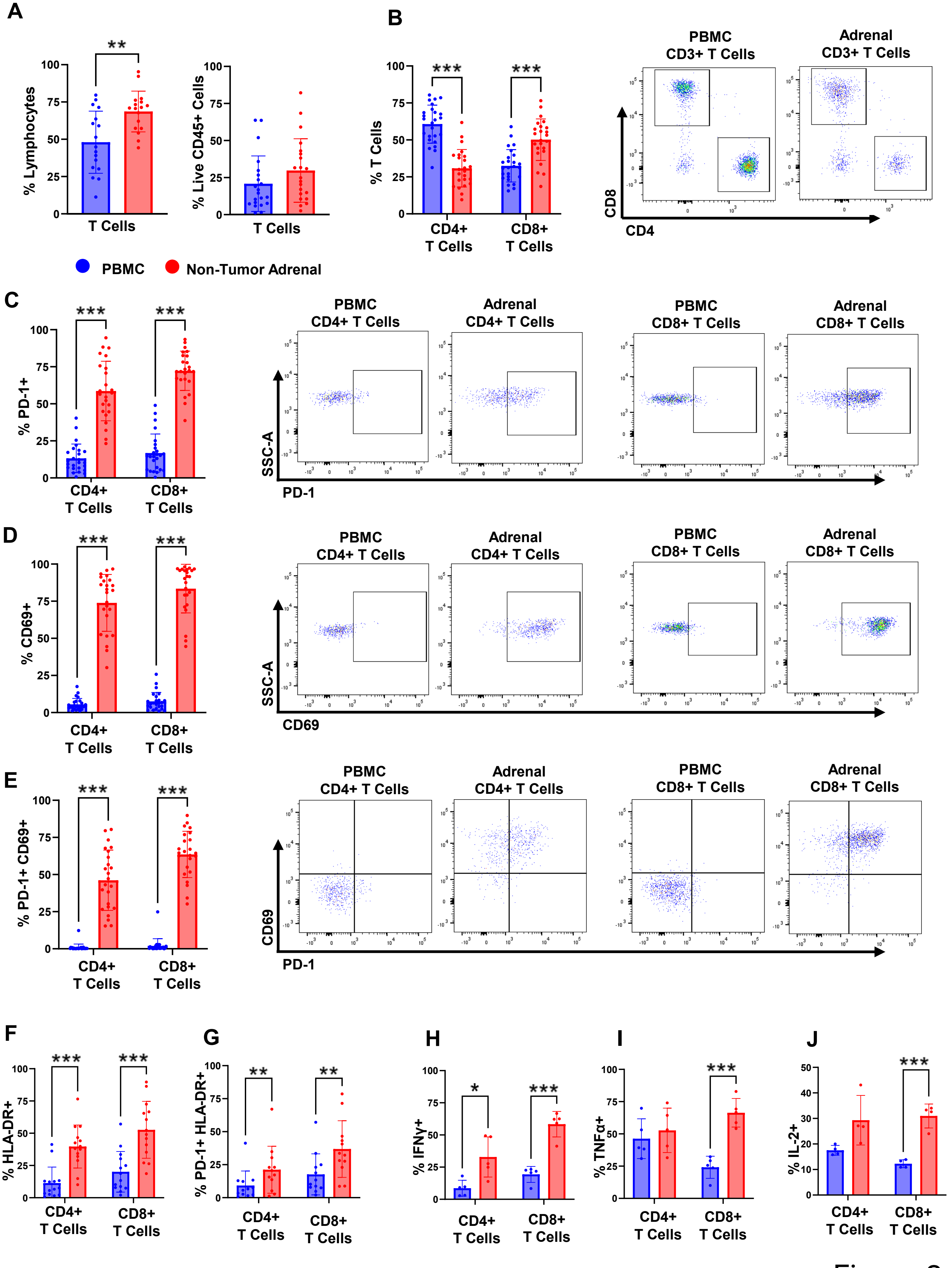

A
% Lymphocytes
**
T Cells
% Live CD45+ Cells
T Cells
PBMC
Non-Tumor Adrenal
B
% T Cells
***
CD4+ T Cells
CD8+ T Cells
PBMC CD3+ T Cells
Adrenal CD3+ T Cells
CD8
CD4
C
% PD-1+
PBMC CD4+ T Cells
Adrenal CD4+ T Cells
PBMC CD8+ T Cells
Adrenal CD8+ T Cells
SSC-A
PD-1
D
% CD69+
CD69
E
% PD-1+ CD69+
F
% HLA-DR+
G
% PD-1+ HLA-DR+
H
% IFNγ+
*
I
% TNFα+
J
% IL-2+

Figure 3

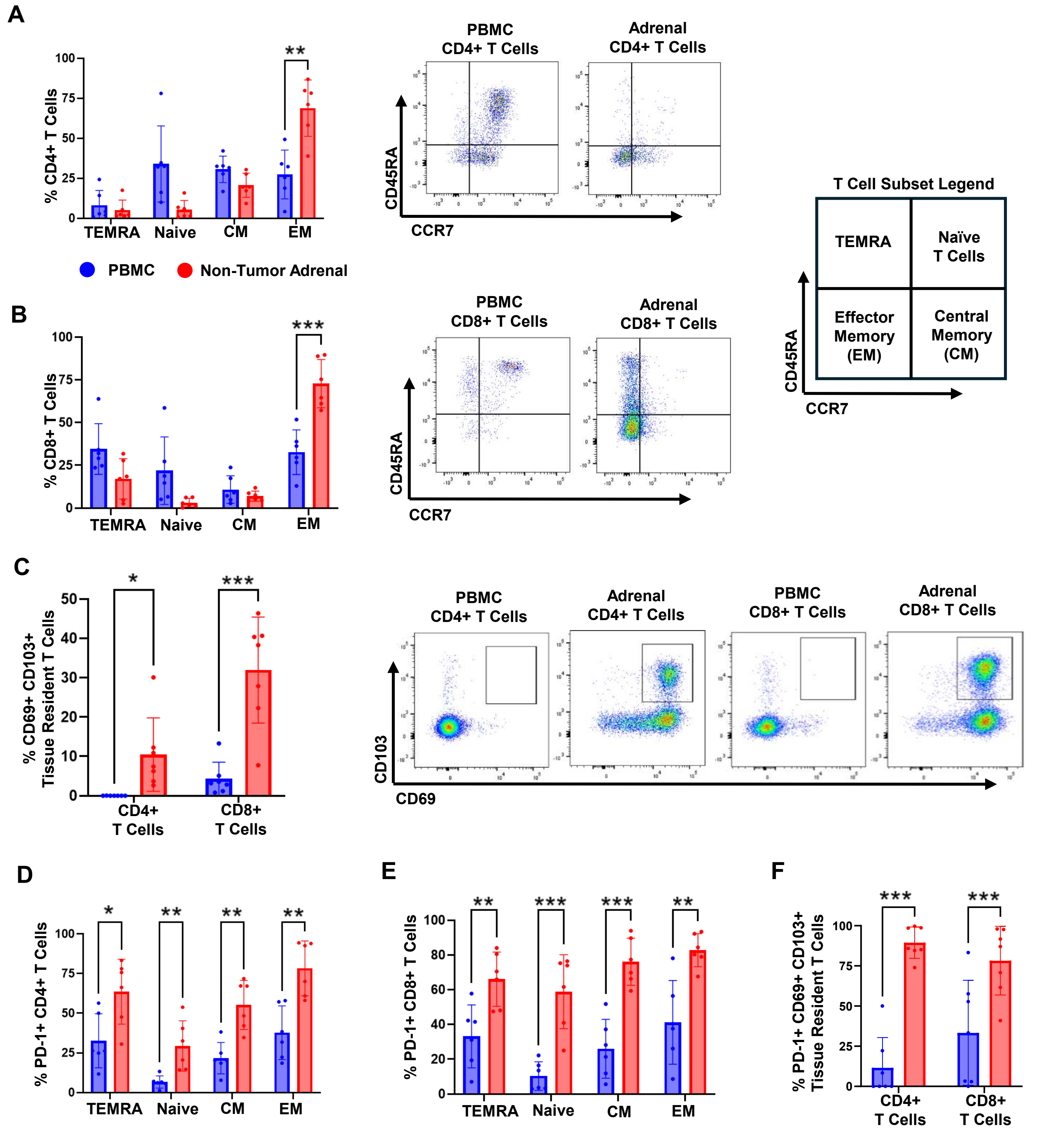

A
% CD4+ T Cells
TEMRA
Naive
CM
EM
PBMC
Non-Tumor Adrenal
PBMC CD4+ T Cells
Adrenal CD4+ T Cells
CD45RA
CCR7
T Cell Subset Legend
TEMRA
Naïve T Cells
Effector Memory (EM)
Central Memory (CM)
B
% CD8+ T Cells
PBMC CD8+ T Cells
Adrenal CD8+ T Cells
C
% CD69+ CD103+ Tissue Resident T Cells
CD4+ T Cells
CD8+ T Cells
CD103
CD69
D
% PD-1+ CD4+ T Cells
E
% PD-1+ CD8+ T Cells
F
% PD-1+ CD69+ CD103+ Tissue Resident T Cells

Figure 4

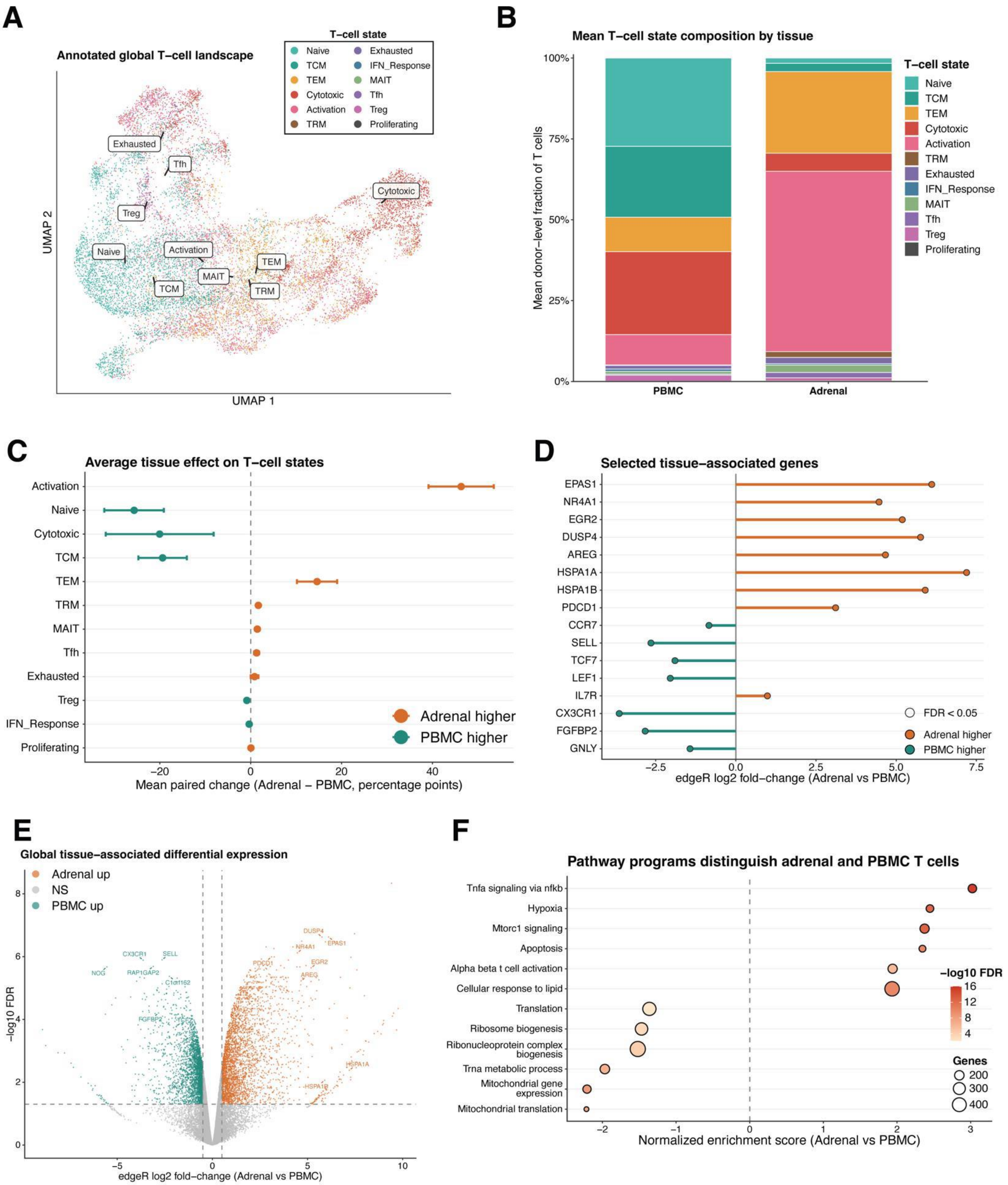

A
Annotated global T−cell landscape
T−cell state
Naive
TCM
TEM
Cytotoxic
Activation
TRM
Exhausted
IFN_Response
MAIT
Tfh
Treg
Proliferating
Exhausted
Tfh
Treg
Naive
Activation
TCM
MAIT
TEM
TRM
Cytotoxic
UMAP 2
UMAP 1
B
Mean T−cell state composition by tissue
Mean donor−level fraction of T cells
100%
75%
50%
25%
0%
PBMC
Adrenal
C
Average tissue effect on T−cell states
Activation
Naive
Cytotoxic
TCM
TEM
TRM
MAIT
Tfh
Exhausted
Treg
IFN_Response
Proliferating
Adrenal higher
PBMC higher
−20
0
20
40
Mean paired change (Adrenal − PBMC, percentage points)
D
Selected tissue−associated genes
EPAS1
NR4A1
EGR2
DUSP4
AREG
HSPA1A
HSPA1B
PDCD1
CCR7
SELL
TCF7
LEF1
IL7R
CX3CR1
FGFBP2
GNLY
FDR < 0.05
Adrenal higher
PBMC higher
−2.5
0.0
2.5
5.0
7.5
edgeR log2 fold−change (Adrenal vs PBMC)
E
Global tissue−associated differential expression
Adrenal up
NS
PBMC up
−log10 FDR
edgeR log2 fold−change (Adrenal vs PBMC)
F
Pathway programs distinguish adrenal and PBMC T cells
Tnfa signaling via nfkb
Hypoxia
Mtorc1 signaling
Apoptosis
Alpha beta t cell activation
Cellular response to lipid
Translation
Ribosome biogenesis
Ribonucleoprotein complex biogenesis
Trna metabolic process
Mitochondrial gene expression
Mitochondrial translation
−log10 FDR
16
12
8
4
Genes
200
300
400
−2
−1
0
1
2
3
Normalized enrichment score (Adrenal vs PBMC)

Figure 5

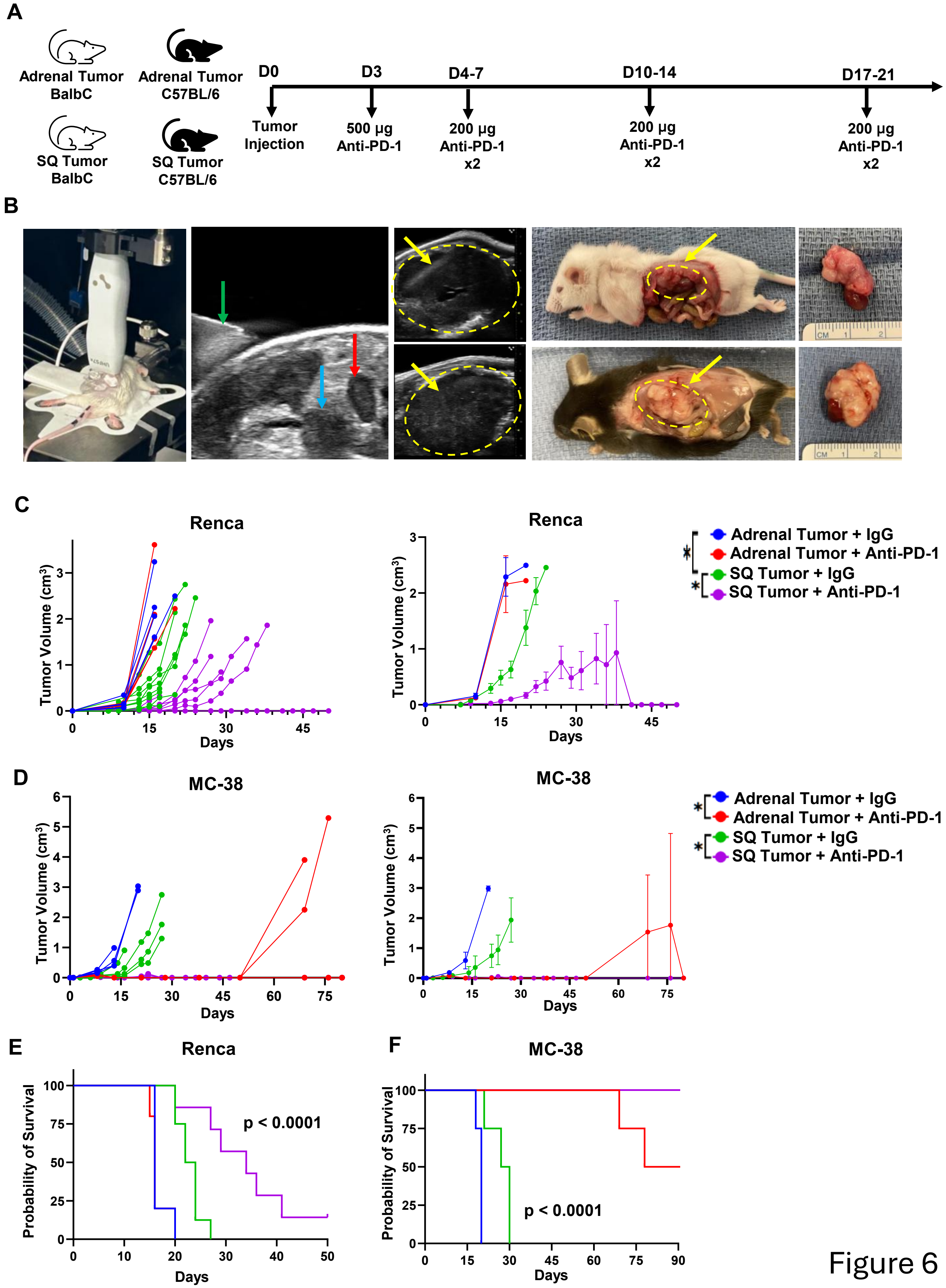

A
Adrenal Tumor BalbC
Adrenal Tumor C57BL/6
SQ Tumor BalbC
SQ Tumor C57BL/6
D0
D3
D4-7
D10-14
D17-21
Tumor Injection
500 µg Anti-PD-1
200 µg Anti-PD-1 x2
200 µg Anti-PD-1 x2
200 µg Anti-PD-1 x2
B
C
Renca
Tumor Volume (cm³)
Days
Adrenal Tumor + IgG
Adrenal Tumor + Anti-PD-1
SQ Tumor + IgG
SQ Tumor + Anti-PD-1
D
MC-38
E
Renca
Probability of Survival
p < 0.0001
F
MC-38
p < 0.0001

Figure 6

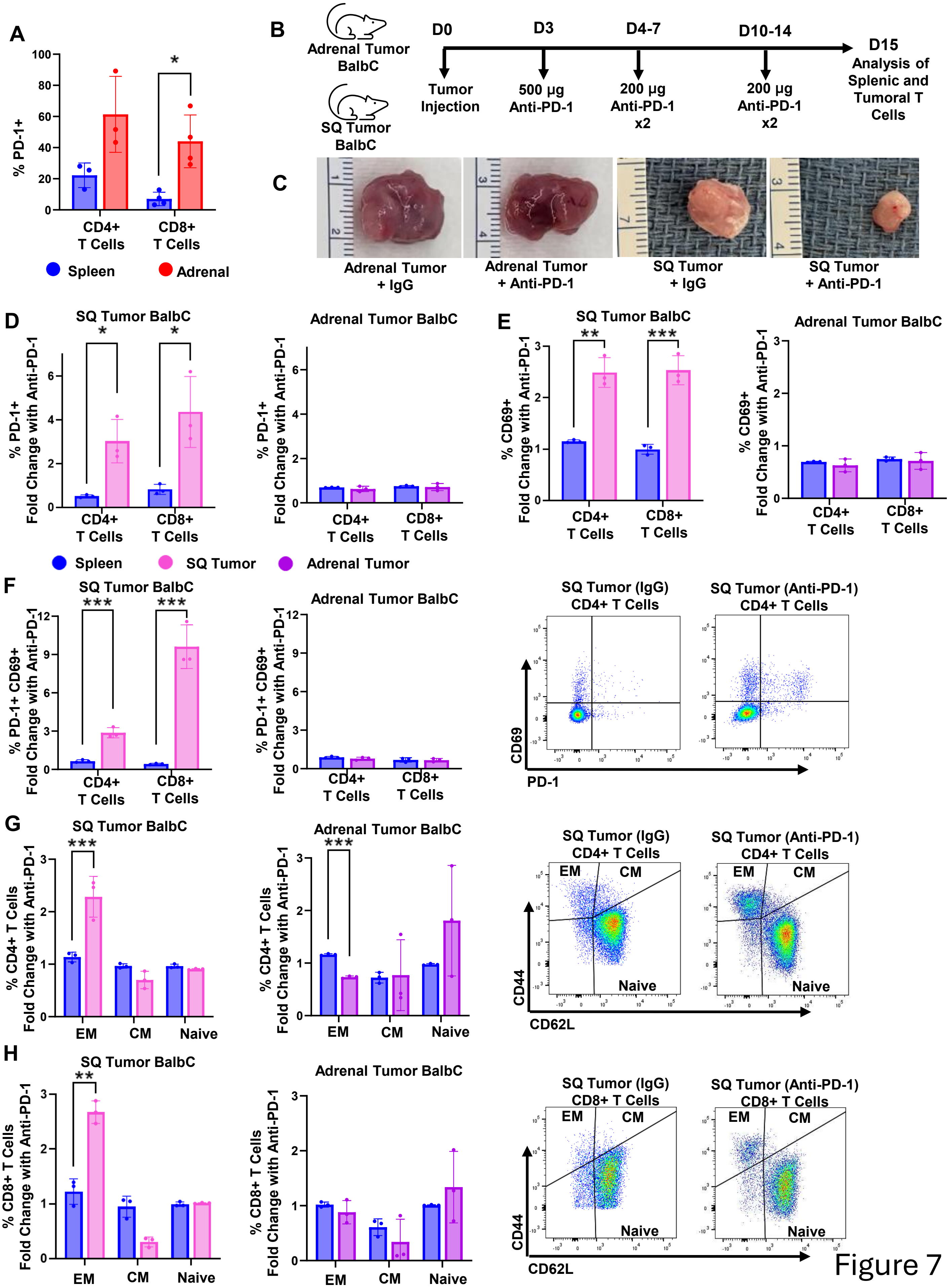

A
% PD-1+
CD4+ T Cells
CD8+ T Cells
Spleen
Adrenal
B
Adrenal Tumor BalbC
SQ Tumor BalbC
D0
D3
D4-7
D10-14
D15
Tumor Injection
500 µg Anti-PD-1
200 µg Anti-PD-1 x2
200 µg Anti-PD-1 x2
Analysis of Splenic and Tumoral T Cells
C
Adrenal Tumor + IgG
Adrenal Tumor + Anti-PD-1
SQ Tumor + IgG
SQ Tumor + Anti-PD-1
D
SQ Tumor BalbC
Adrenal Tumor BalbC
% PD-1+ Fold Change with Anti-PD-1
E
% CD69+ Fold Change with Anti-PD-1
Spleen
SQ Tumor
Adrenal Tumor
F
% PD-1+ CD69+ Fold Change with Anti-PD-1
SQ Tumor (IgG) CD4+ T Cells
SQ Tumor (Anti-PD-1) CD4+ T Cells
CD69
PD-1
G
% CD4+ T Cells Fold Change with Anti-PD-1
EM
CM
Naive
CD44
CD62L
H
% CD8+ T Cells Fold Change with Anti-PD-1
SQ Tumor (IgG) CD8+ T Cells
SQ Tumor (Anti-PD-1) CD8+ T Cells

Figure 7

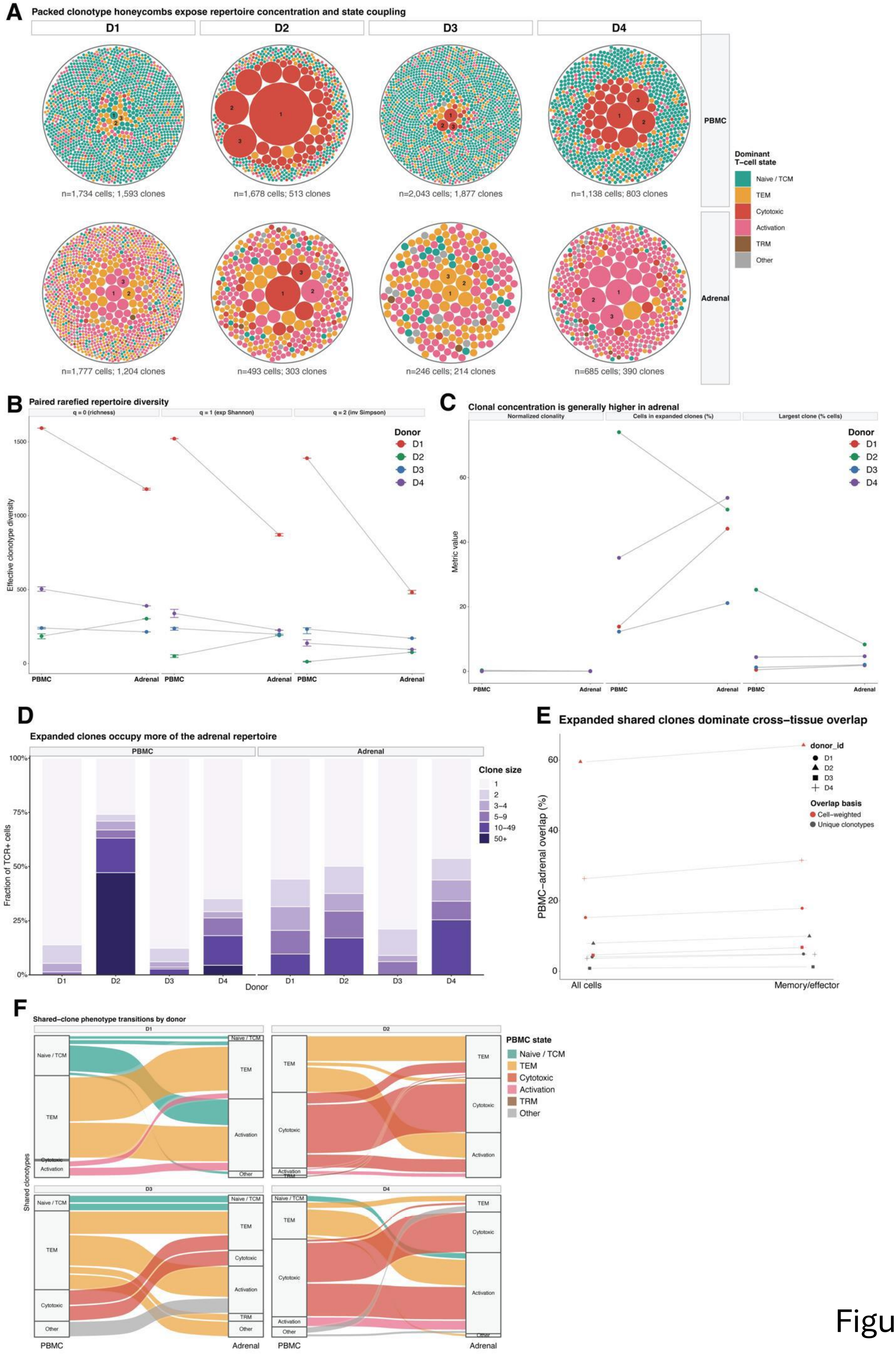

A
Packed clonotype honeycombs expose repertoire concentration and state coupling
D1
D2
D3
D4
PBMC
Adrenal
n=1,734 cells; 1,593 clones
n=1,678 cells; 513 clones
n=2,043 cells; 1,877 clones
n=1,138 cells; 803 clones
n=1,777 cells; 1,204 clones
n=493 cells; 303 clones
n=246 cells; 214 clones
n=685 cells; 390 clones
Dominant T−cell state
Naive / TCM
TEM
Cytotoxic
Activation
TRM
Other
B
Paired rarefied repertoire diversity
q = 0 (richness)
q = 1 (exp Shannon)
q = 2 (inv Simpson)
Effective clonotype diversity
Donor
C
Clonal concentration is generally higher in adrenal
Normalized clonality
Cells in expanded clones (%)
Largest clone (% cells)
Metric value
D
Expanded clones occupy more of the adrenal repertoire
Fraction of TCR+ cells
Clone size
1
2
3–4
5–9
10–49
50+
Donor
E
Expanded shared clones dominate cross−tissue overlap
PBMC−adrenal overlap (%)
donor_id
Overlap basis
Cell−weighted
Unique clonotypes
All cells
Memory/effector
F
Shared−clone phenotype transitions by donor
Shared clonotypes
PBMC state
PBMC
Adrenal

Figure 8

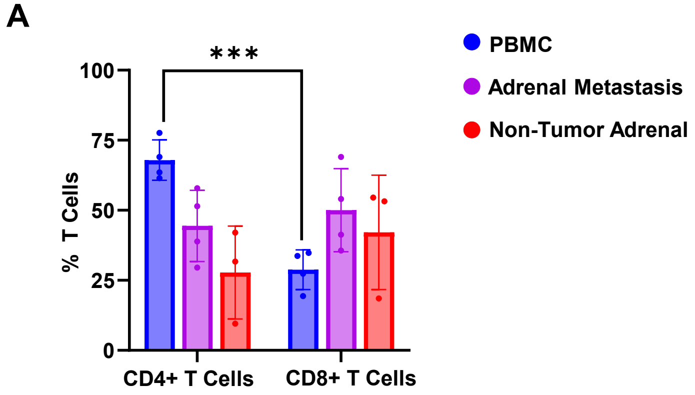

Supplemental Figure 1

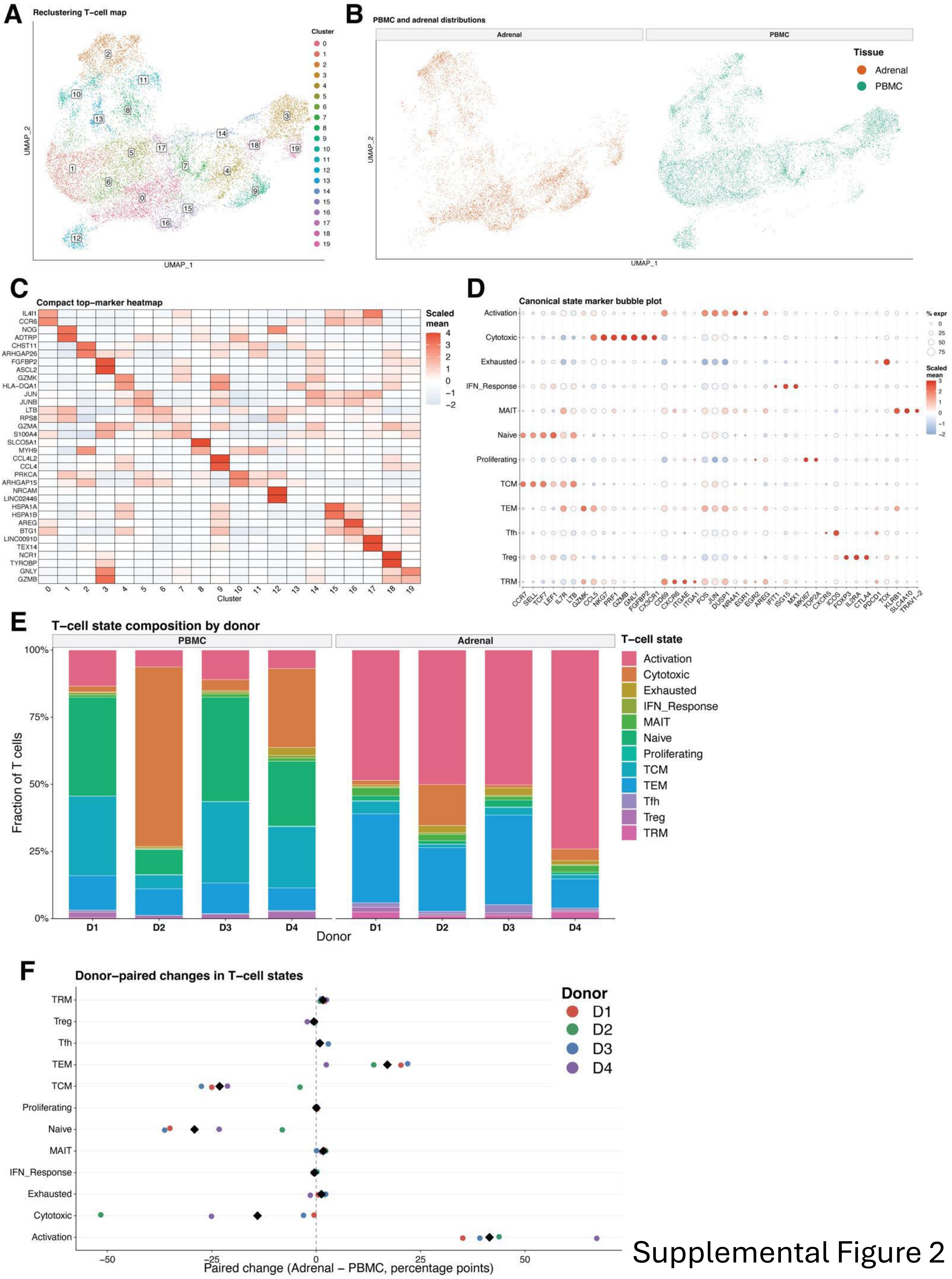

Supplemental Figure 2

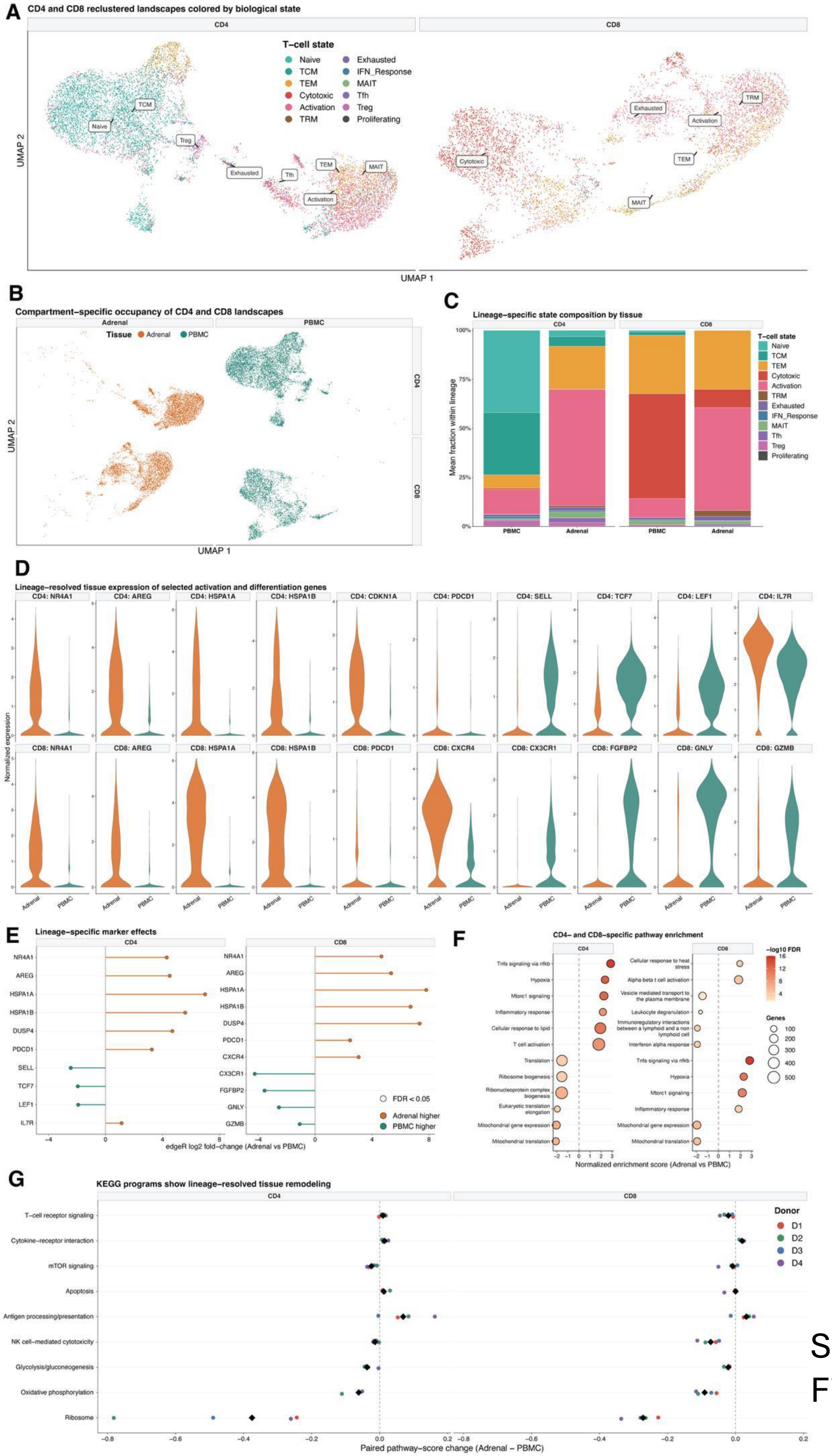

Supplemental Figure 3

Supplemental Figure 4
